# Tissue engineering RPE sheet derived from hiPSC-RPE cell spheroids supplemented with Y-27632 and RepSox

**DOI:** 10.1186/s13036-024-00405-8

**Published:** 2024-01-16

**Authors:** Wenxuan Wang, Tingting Yang, Sihui Chen, Liying Liang, Yingxin Wang, Yin Ding, Wei Xiong, Xiuhong Ye, Yonglong Guo, Shuhao Shen, Hang Chen, Jiansu Chen

**Affiliations:** 1 Department of Ophthalmology, The First Affiliated Hospital of Jinan University, Guangzhou, China; 2 Institute of Ophthalmology, Medical College, Jinan University, Guangzhou, China; 3 The University of Hong Kong - Shenzhen Hospital, Shenzhen, China; 4 Key Laboratory for Regenerative Medicine, Ministry of Education, Jinan University, Guangzhou, China; 5 College of Veterinary Medicine, South China Agricultural University, Guangzhou, China; 6 Aier Eye Institute, Changsha, Hunan China

**Keywords:** Retinal pigment epithelium, TGF-β, ROCK, Spheroid, Collagen vitrigel scaffold, Tissue engineering

## Abstract

**Background:**

Retinal pigment epithelium (RPE) cell therapy is a promising way to treat many retinal diseases. However, obtaining transplantable RPE cells is time-consuming and less effective. This study aimed to develop novel strategies for generating engineered RPE patches with physiological characteristics.

**Results:**

Our findings revealed that RPE cells derived from human induced pluripotent stem cells (hiPSCs) successfully self-assembled into spheroids. The RPE spheroids treated with Y27632 and Repsox had increased expression of epithelial markers and RPE-specific genes, along with improved cell viability and barrier function. Transcriptome analysis indicated enhanced cell adhesion and extracellular matrix (ECM) organization in RPE spheroids. These RPE spheroids could be seeded and bioprinted on collagen vitrigel (CV) membranes to construct engineered RPE sheets. Circular RPE patches, obtained by trephining a specific section of the RPE sheet, exhibited abundant microvilli and pigment particles, as well as reduced proliferative capacity and enhanced maturation.

**Conclusions:**

Our study suggests that the supplementation of small molecules and 3D spheroid culture, as well as the bioprinting technique, can be effective methods to promote RPE cultivation and construct engineered RPE sheets, which may support future clinical RPE cell therapy and the development of RPE models for research applications.

**Supplementary Information:**

The online version contains supplementary material available at 10.1186/s13036-024-00405-8.

## Background

The retinal pigment epithelium (RPE) is a highly specialized and polarized single-cell sheet located in the outer retina. RPE cells play a critical role in the visual cycle, light absorption, the photoreceptor outer segments (POS) absorption, formation of the blood-retinal barrier (BRB), and transportation of nutrients and ions [1]. Dysfunctions in the RPE which can lead to disruption of the BRB and photoreceptor cell death, are implicated in many blinding retinal diseases, such as age-related macular degeneration and retinitis pigmentosa [2–4]. Therefore, the therapies targeting RPE are of great importance. A few RPE cell therapies have been conducted utilizing human induced pluripotent stem cells (hiPSCs) or human embryonic stem cells (hESCs) [5–7]. Compared to hESCs, hiPSCs offer the advantage of circumventing issues related to immune rejection, adverse effects associated with immunosuppressants and ethical concerns, making hiPSCs an abundant and accessible cell source for cell therapy [8]. Despite the remarkable progress, the development of clinically approved hiPSC-derived RPE for stem cell therapy remains an ongoing process. Clinical-grade and qualified hiPSC-RPE must exhibit a polarized and mature phenotype, possess a functional barrier and display low proliferative capacity to mitigate the risk of tumorigenicity [9, 10]. Besides, there is a need for improved production efficiency, as the current methods are often time-consuming [11].

Small molecules are known to manipulate cell fate, state, and function in vitro by targeting specific proteins [12]. By controlling duration time and concentration, the manipulation of small molecule compounds can be flexible and convenient. Among these small molecules, Y27632, classified as a rho-associated protein kinase(ROCK) inhibitor, has been reported to promote adhesion, extended passages, anti-apoptosis, pigmentation, and morphological characteristics of hiPSC-RPE [13, 14]. Repsox, also known as 616452, functions as an inhibitor of the transforming growth factor-beta (TGF-β) receptor and is widely used in iPSC reprogramming and lineage reprogramming due to its ability to replace Sox2 and c-Myc by inhibiting TGF-β signaling pathways [15, 16]. Both Y27632 and Repsox are integral components of small molecule cocktails utilized in the direct reprogramming of human astrocytes into early neuroectodermal cells [17]. However, the combined impact of Y27632 and Repsox on stem-cell derived RPE cells is explored little. These small molecules have been reported to intervene in epithelial-mesenchymal transition (EMT) in an EMT model derived from hESC-RPE [18]. Nevertheless, the effects of Y27632 and Repsox on hiPSC-RPE cells remain to be elucidated.

Three-dimensional (3D) spheroid cell culture is a popular method for tissue engineering [19]. Compared to conventional 2D cell culture which may induce genetic and epigenetic alterations, as well as characteristic changes in cells, 3D spheroid culture offers a more physiologically relevant environment for cell growth [20]. Spheroid culture facilitates robust cell-cell interactions and cell-extracellular matrix (ECM) interactions for the cultured cells [21]. Our previous research demonstrated enhanced stemness in spheroid culture for corneal stromal cells and corneal endothelial cells [22, 23]. In regards to research on hiPSC-RPE cells, our group also observed enhanced RPE characteristics resulting from spheroid culture [24]. Moreover, spheroids have been recognized as more amenable to engineering and bioprinting than dissociated cells [25]. Through spheroid bioprinting, 3D bioprinted cardiac patches have been successfully constructed [26]. Therefore, it is viable to use hiPSC-RPE spheroids and bioprinting techniques for cultivating qualified RPE cells and constructing engineered hiPSC-RPE patches.

In the current study, we first investigated the effects of combining a ROCK inhibitor (Y27632) and a TGF-β inhibitor (Repsox) on hiPSC-RPE and hiPSC-RPE spheroids, providing insights into the mechanisms of the changes brought by spheroid culture in hiPSC-RPE cells. Then, for the first time, we used small-molecule cocktails comprised of Y27632 and Repsox, 3D spheroid culture technique and the natural scaffold from type I collagen to construct engineered hiPSC-RPE sheets and we are the first to describe a selection of RPE patches with enhanced physiological characteristics. Moreover, we constructed engineered RPE sheets by spheroid bioprinting, which provides a valuable method for RPE model construction and cell therapy.

## Results

### Generation and identification of hiPSC-derived RPE cells

**Fig. 1:**
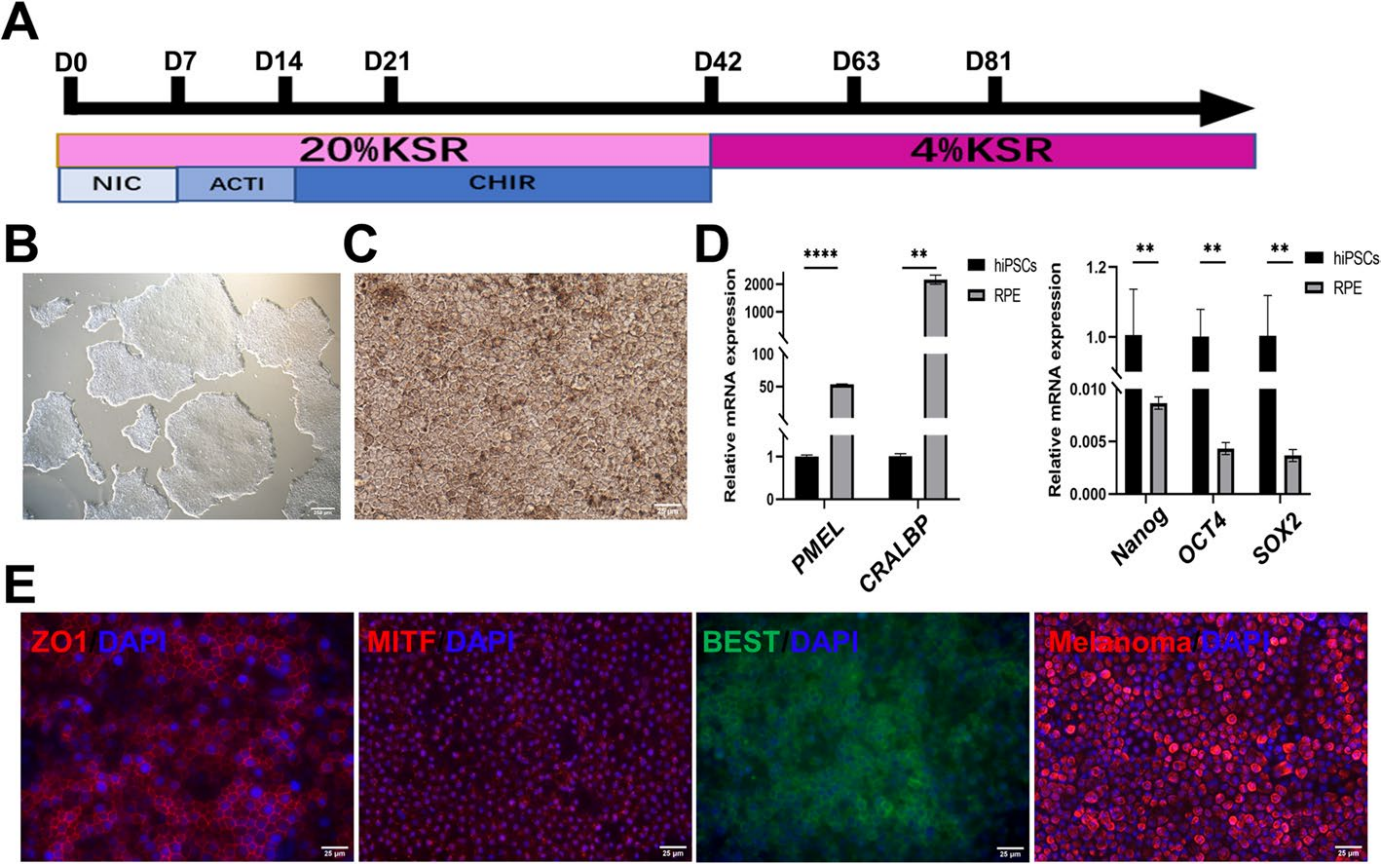
Process of differentiation from human induced pluripotent stem cells (hiPSCs) into retinal pigment epithelial (RPE) cells. **A** Diagram of differentiation from hiPSCs into RPE cells. **B** Morphological observation of hiPSCs. Scale bar 250 μm. **C** Morphological observation of RPE cells. Scale bar 25 μm. **D** Quantitative real-time polymerase chain reaction (RT-qPCR) results of mRNA expression for *Nanog*, *OCT4*, *SOX2*, *CRALBP*, *PMEL*. **E** Immunofluorescence staining of ZO1, MITF, BEST, Melanoma in RPE cells. Scale bar 25 μm. Mean ± SD (**p < 0.01; ****p < 0.0001; *n* = 3)

The human iPSC cells were induced to differentiate into hiPSC-RPE cells following the protocol previously described by Florian Regent et al. [27] (Fig. 1A). The human iPSC cell line exhibited colony growth (Fig. 1B). After 42 days of differentiation, hiPSC-RPE cells were harvested and cultured for additional 3 weeks for further identification. Bright-field microscopy and immunofluorescence staining were performed to observe the hiPSC-RPE cells. The differentiated hiPSC-RPE cells showed growing pigmentation with culture time increasing and formed a cobblestone-like shape eventually (Fig. 1C). Quantitative real-time polymerase chain reaction (RT-qPCR) analysis revealed a significant reduction in the mRNA expression levels of pluripotent genes in the RPE cells derived from the human iPSC cell line (Fig. 1D). Conversely, the expression levels of *CRALBP* (cellular retinaldehyde-binding protein, a visual cycle marker) and *PMEL* (also named melanoma) were significantly increased in hiPSC-RPE cells (Fig. 1D). These cells also exhibited positive expression of tight junction marker ZO1, ion transportation marker BEST, melanogenesis factor Melanoma, and microphthalmia-associated transcription factor MITF (Fig. 1E). The ratio of MITF positive cells is 99.1% (Fig. S1). These results confirmed the successful differentiation and collection of hiPSC-RPE cells.

### Enhancement of adherence for dissociated hiPSC-RPE cells supplemented with Y-27632 and RepSox

**Fig. 2:**
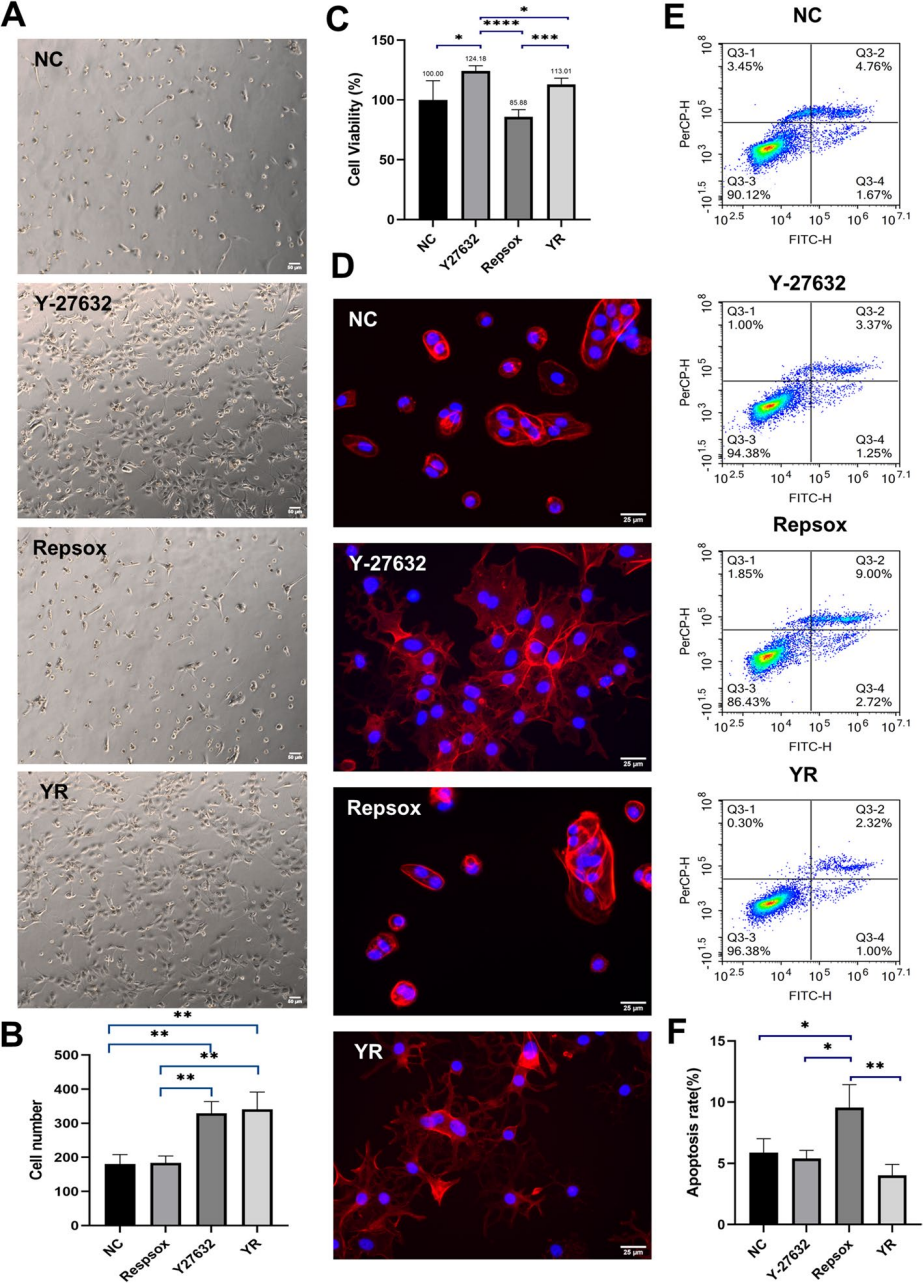
Cell viability, apoptosis rate, and adherence of RPE cells supplemented with Y27632 and Repsox. **A**, **B** Observation and analysis of adherent RPE cells treated with Y27632 and Repsox. Scale bar 50 μm. **C** Cell viability of RPE cells supplemented with Y27632 and Repsox. **D** Immunofluorescence staining of F-actin in RPE cells supplemented with Y27632 and Repsox. Scale bar 25 μm. **E**, **F** Apoptosis analysis of RPE cells treated with Y27632 and Repsox. Mean ± SD (*p < 0.05; **p < 0.01; ***p < 0.001; ****p < 0.0001; *n* = 3)

Next, we investigated the effects of Y27632 and Repsox on hiPSC-RPE cells in order to obtain high-quality cells. The differentiated hiPSC-RPE cells were seeded at a same density and distributed into four groups: the control group (cultured without additional compounds), Y27632 group (treated with Y27632), Repsox group (treated with Repsox), and YR group (treated with Repsox + Y27632). Both Y27632 and Repsox were used at a concentration of 10 μM and added during cell passage. After 24 h of culture, we observed that the number of adherent cells was significantly higher in the Y27632 group (329 ± 34.70) and YR group (340.67 ± 50.64) compared to the control group (180.67 ± 27.54) and Repsox group (183.67 ± 20.43) (Fig. 2A). Statistical significance was observed in the following comparisons: control group vs Y27632 group (*P* < 0.01), control group vs YR group (*P* < 0.01), Repsox group vs Y27632 group (*P* < 0.01), Repsox group vs YR group (*P* < 0.01) (Fig. 2B). After that, we seeded hiPSC-RPE cells at a same density and cultured for 24 h. Then we treated these small molecules for another 24 h and conducted CCK8 test. The findings from the CCK8 assays showed that the cell viability in the Y27632 group (124.18 ± 4.43%) was significantly higher than that in the control group (100 ± 16.10%) (*P* < 0.05), Repsox group (85.88 ± 5.88%) (*P* < 0.0001), and YR group (113.01 ± 5.19%) (*P* < 0.05) (Fig. 2C). Additionally, the cell viability in the YR group was higher than that in the Repsox group (*P* < 0.001) (Fig. 2C). Immunofluorescence staining of phalloidine revealed that the hiPSC-RPE cells supplemented with Y27632 and Repsox + Y27632 were anchored onto culture plates with F-actin structures resembling tentacles (Fig. 2D). In contrast, cells in the control group and the Repsox group displayed a round shape (Fig. 2D). These results indicate that the addition of Repsox alone does not enhance the adherence and cell viability of hiPSC-RPE cells, while the inclusion of Y27632 improves the adherence and viability of hiPSC-RPE cells during seeding, which may be attributed to alterations in F-actin assembly and distribution. Moreover, the YR group also improves the cell adherence of hiPSC-RPE cells and the positive effects are mainly due to Y27632.

To further investigate the impact of Y-27632 and RepSox on hiPSC-RPE cells during cell passage, we dissociated hiPSC-RPE cells and exposed them to Y27632, Repsox, and Repsox + Y27632 for 2 h. Subsequently, we analyzed the apoptosis rate of these cells. The results indicated that the apoptosis rate in the Repsox group (9.57 ± 1.87%) is higher than that in the control group (5.88 ± 1.14%) (*P* < 0.05), the Y27632 group (5.39 ± 0.67%) (*P* < 0.05), YR group (4.03 ± 0.89%) (*P* < 0.01) (Fig. 2E, F). In summary, both Y27632 and Y27632 + Repsox contributed to the improved adherence and survival of hiPSC-RPE cells. Notably, the cells treated with Y27632 exhibited superior cell viability.

### Changes of hiPSC-RPE cell characteristics supplemented with Y-27632 and RepSox

**Fig. 3:**
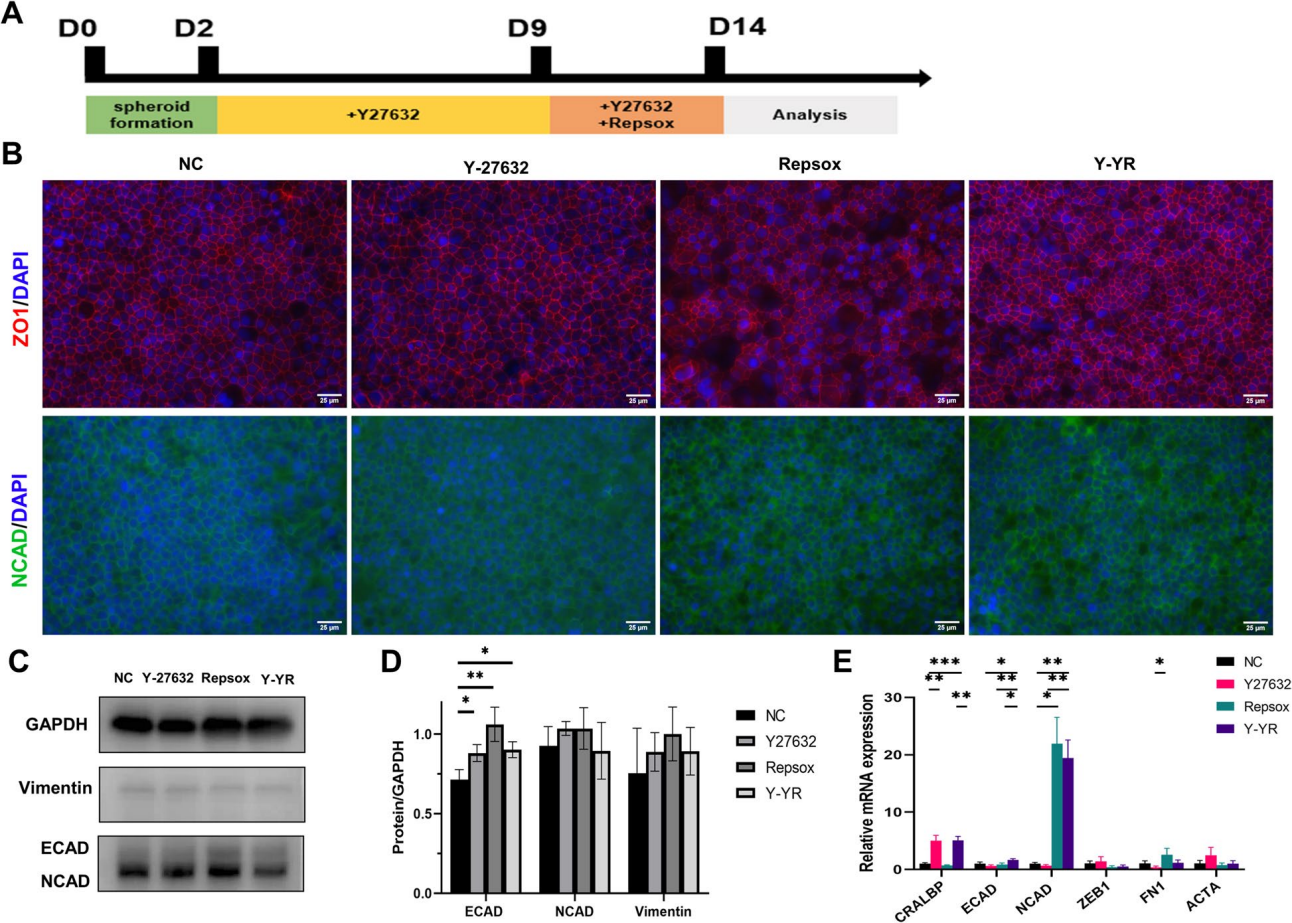
Characterization of RPE-related gene and protein expression in the control group, Y27632 group, Repsox group, and Y-YR group (treated with Y27632 and Y27632 + Repsox sequentially). **A** Diagram of Y27632 and Repsox treatment in the Y-YR group. **B** Immunofluorescence staining of ZO1, NCAD in four groups. Scale bar 25 μm. **C**, **D** Western blot images and analysis of ECAD, NCAD, and Vimentin expression in four groups. **E** RT-qPCR results of mRNA expression for *CRALBP*, *ECAD*, *NCAD*, *ZEB1*, *FN1*,and *ACTA* in four groups. Mean ± SD (*p < 0.05; **p < 0.01; ***p < 0.001; *n* = 3)

To gain a deeper understanding of the long-term effects of these small molecules on hiPSC-RPE cells, we divided the RPE cells into four groups: the control group, Y27632 group (treated with Y27632), Repsox group (treated with Repsox), and Y-YR group, in which we added Y27632 for 10 days and then supplemented with Y27632 + Repsox for another 5 days (Fig. 3A). Immunofluorescence staining was conducted on day 15. The staining results revealed that the presence of ZO1 and N-cadherin in all groups. The hiPSC-RPE cells treated with Repsox displayed a partial disruption of the tight junction marker ZO1, while the other three groups exhibited a more regular ZO1 expression (Fig. 3B). Western blot images and analysis showed significant differences were observed in E-cadherin expression and the differences existed between the control group and Y27632 group (*P* < 0.05), the control group and Repsox group (*P* < 0.05), and the control group and Y-YR group (*P* < 0.05) (Fig. 3C, D). Subsequently, we conducted RT-qPCR and the results of RT-qPCR revealed significant up-regulation in the RNA expression of *ECAD* (E-cadherin) and *NCAD* (N-cadherin) in the Repsox group and Y-YR group (Fig. 3E), consistent with the western blot results. The addition of Y27632 and Y-YR significantly up-regulated the expression of *CRALBP*, while the addition of Repsox induced a significant up-regulation of *FN1* (Fibronectin, a marker associated with EMT) (Fig. 3E). The karyotype analysis of hiPSC-RPE cells treated with Y-YR showed no obvious chromosomal abnormalities (Fig. S2). In summary, our findings suggest that the addition of Repsox may not benefit hiPSC-RPE cells, whereas treatment with Y27632 and Y-YR could enhance the characteristics of hiPSC-RPE cells.

### Promotion of hiPSC-RPE spheroid formation and characteristics added with Y-27632 and RepSox

**Fig. 4:**
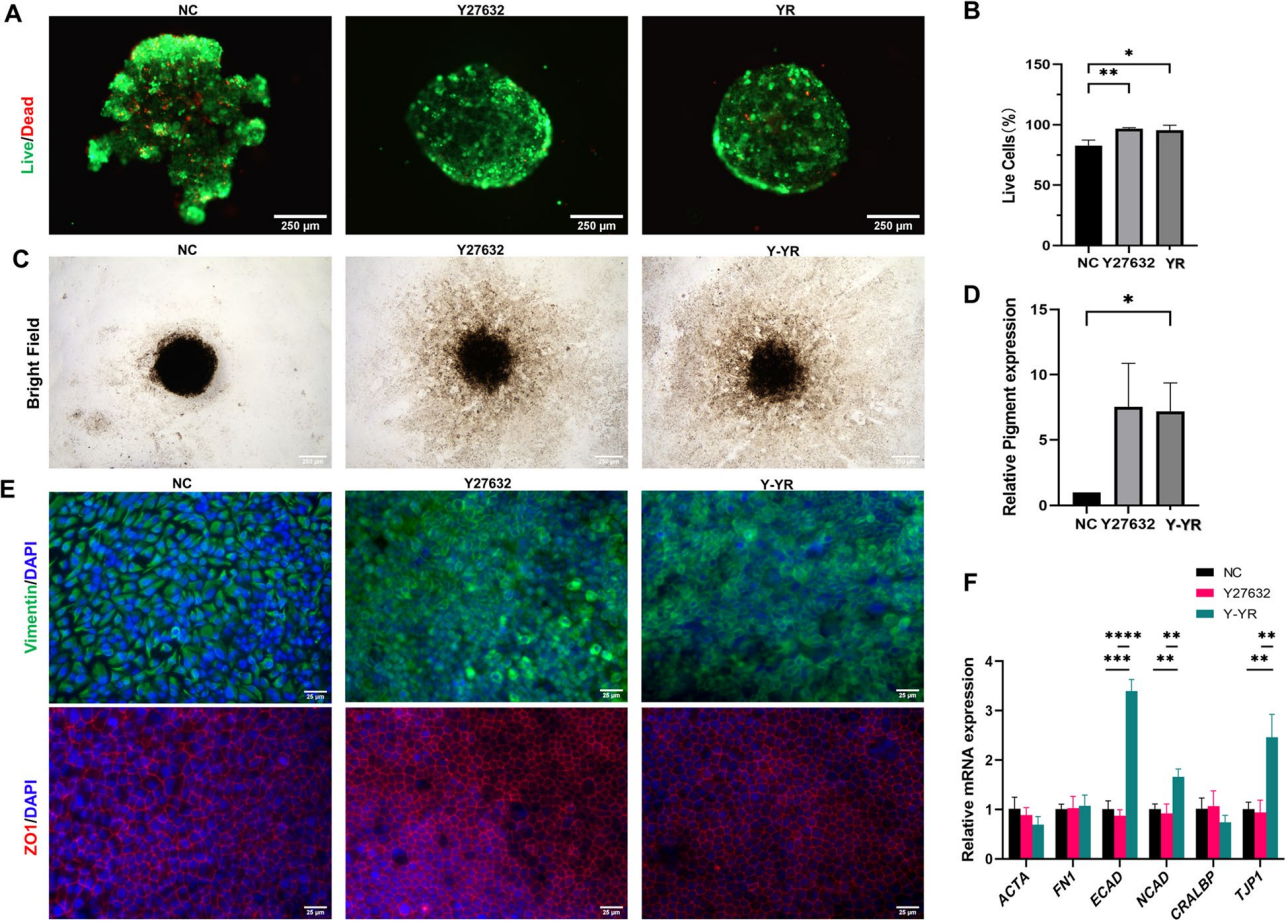
Morphology and gene expression of RPE spheroids in the control group, Y27632 group and Y-YR group (treated with Y27632 and Y27632 + Repsox sequentially). **A**, **B** Live and dead assay of RPE spheroids in three groups. Scale bar 250 μm. **C**, **D** Analysis and bright-field images of pigment area in RPE spheroids. Scale bar 250 μm. **E** Immunofluorescence staining of ZO1, Vimentin in three groups. Scale bar 25 μm. **F** RT-qPCR results of mRNA expression for *CRALBP, ECAD, NCAD, TJP1, FN1, ACTA* in three groups. Mean ± SD (*p < 0.05; **p < 0.01; ***p < 0.001; ****p < 0.0001; *n* = 3)

The hiPSC-RPE cells could aggregate into cell spheroids in agarose micro-molds (Fig. S3). To investigate the effects of Y27632 and Repsox on the formation of hiPSC-RPE cell spheroids, we respectively seeded hiPSC-RPE cells into agarose micromolds with Y27632, Repsox, and Y27632 + Repsox. After 48 h, hiPSC-RPE spheroids were collected and stained with a live/dead cell imaging kit. In the Y27632 group and Y27632 + Repsox group, most hiPSC-RPE cells were alive and successfully formed spheroids. Comparatively, in the absence of these small molecules, the cells could not form cohesive spheroids and simply aggregated irregularly (Fig. 4A). Compared to the control group (82.85 ± 4.42%), the proportion of live cells increased significantly in the Y27632 group (96.87 ± 0.75%) (*P* < 0.01) and YR group (95.48 ± 4.16%) (*P* < 0.05) (Fig. 4B). These findings demonstrate that Y27632 and Y27632 + Repsox can promote the formation and viability of hiPSC-RPE cell spheroids.

Next, we examined the prolonged impact of Y27632 and Y27632 + Repsox on hiPSC-RPE cell spheroids. At first, we seeded hiPSC-RPE spheroids on plates and treated them with Y27632 and Y-YR, as described above. On day 15, bright field microscopy images revealed that the cell spheroids in the control group exhibited limited expansion with pigmented hiPSC-RPE cells. In contrast, the addition of Y27632 and Y-YR prompted hiPSC-RPE cell spheroids to expand radially with an increased population of pigmented hiPSC-RPE cells (Fig. 4C). The results indicated that the positive effects of cell expansion are mainly due to Y27632. Quantitative analysis of the pigmented area showed a significant difference between the YR group and the control group (*P* < 0.05) (Fig. 4D). Immunostaining results displayed consistent expression of vimentin and ZO1 in all groups. The cells treated with Y27632 and Y-YR showed a more compact polygonal morphology and a more regular ZO1 expression compared to non-treated cells (Fig. 4E). In addition, vimentin (a marker of EMT) was expressed in different patterns among these groups. In the control group, vimentin was evenly distributed and highly expressed in the cytoplasm,while in the Y27632 and Y-YR groups, it was distributed closer to the cell membrane (Fig. 4E). Furthermore, RT-qPCR analysis demonstrated that *ECAD*, *NCAD*, and *TJP1* were significantly up-regulated in the Y27632 + Repsox group compared to the control group and Y27632 group (Fig. 4F). Collectively, the above results indicate that Y27632 and Y-YR can facilitate the expansion of RPE spheroids. The Y-YR treatment can promote an epithelial phenotype in hiPSC-RPE cells and hiPSC-RPE spheroids.

### Better RPE characteristics and barrier function using spheroid culture than dissociated cell culture when supplemented with Y-27632 and RepSox

**Fig. 5:**
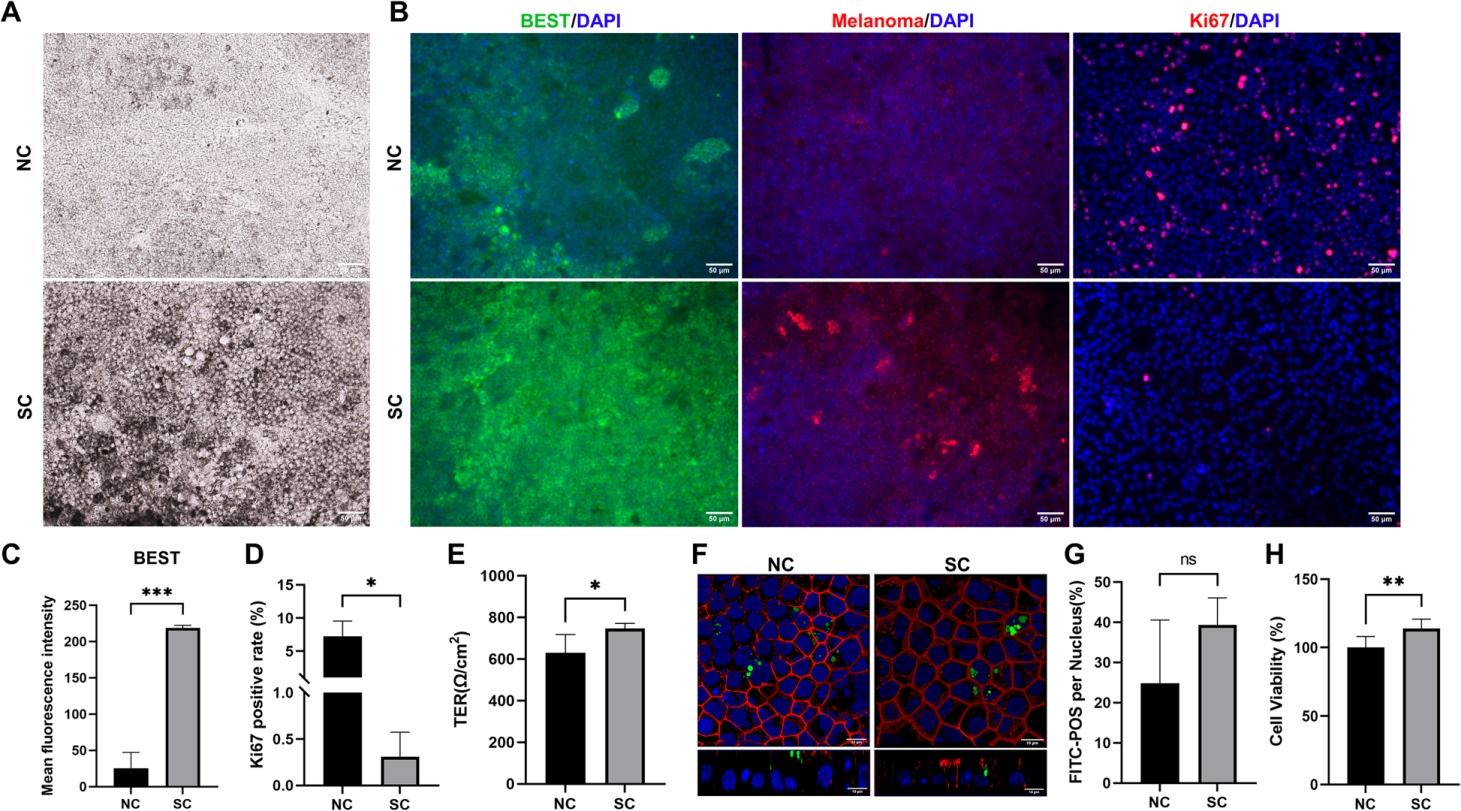
Enhanced RPE characteristics and barrier function using spheroid culture and Y-YR supplementation. **A** Morphological observation of RPE cells in the normal culture (NC) and spheroid culture (SC) group. Scale bar 250 μm. **B**, **C**, **D** Immunofluorescence staining and analysis of BEST, Melanoma and Ki67 in both groups. Scale bar 250 μm. **E** Transepithelial electrical resistance (TER) assay in both groups. **F**, **G** Photoreceptor outer segments (POS) phagocytosis test and analysis in both groups. Scale bar 10 μm. **H** Cell viability of RPE cells in both groups. Mean ± SD (*p < 0.05; **p < 0.01; ***p < 0.001; *n* = 3 ~ 5)

3D spheroidal cell culture is reported to maintain cellular phenotype and promote cell-cell interaction and cell-ECM interaction in some types of cells [28]. To further investigate the benefits of hiPSC-RPE cell spheroids, the RPE spheroids and dispersed RPE cells were seeded onto plates and cultured with Y-YR. Then, we compared the cells in spheroid culture (SC) group with the cells cultured in the 2D normal culture (NC) group after a 2-week culture. On day 15, hiPSC-RPE cells expanded from spheroids, and the cells adjacent to RPE spheroids showed a greater abundance of pigment granules, compared to conventional 2D culture (Fig. 5A). The expression of Melanoma and BEST was observed in both groups (Fig. 5B). Immunofluorescence staining of the cells in the region close to hiPSC-RPE spheroids displayed a higher expression of BEST (a characteristic marker for RPE cells) and lower expression of Ki67 (Fig. 5B). The differences were statistically significant (Fig. 5C, D). Moreover, the transepithelial electrical resistance (TER) assay and POS phagocytosis test were conducted to evaluate the barrier function and phagocytic function of hiPSC-RPE cells. The results from the Z-stack confocal micrographs showed that both groups had the ability of phagocytosis (Fig. 5F), with no statistically significant differences between them (Fig. 5G). The TER values of cells derived from hiPSC-RPE spheroids (746.62 ± 24.65Ω/cm^2^) were higher than the cells cultured in the conventional 2D method (629.84 ± 87.81Ω/cm^2^) and the difference was statistically significant (*P* < 0.05) (Fig. 5E). Additionally, the results of the CCK8 assay revealed that the viability of cells derived from spheroids (114.10 ± 6.70%) was higher than that of cells cultured using the 2D method (100 ± 8.18%) (*P* < 0.01) (Fig. 5H). Altogether, these data suggest that spheroidal culture can enhance RPE characteristics and the barrier function of hiPSC-RPE cells with the optimal hiPSC-RPE cells located adjacent to RPE spheroids.

### Transcriptome analysis for hiPSC-RPE spheroid and dissociated cell culture when supplemented with Y-27632 and RepSox

**Fig. 6:**
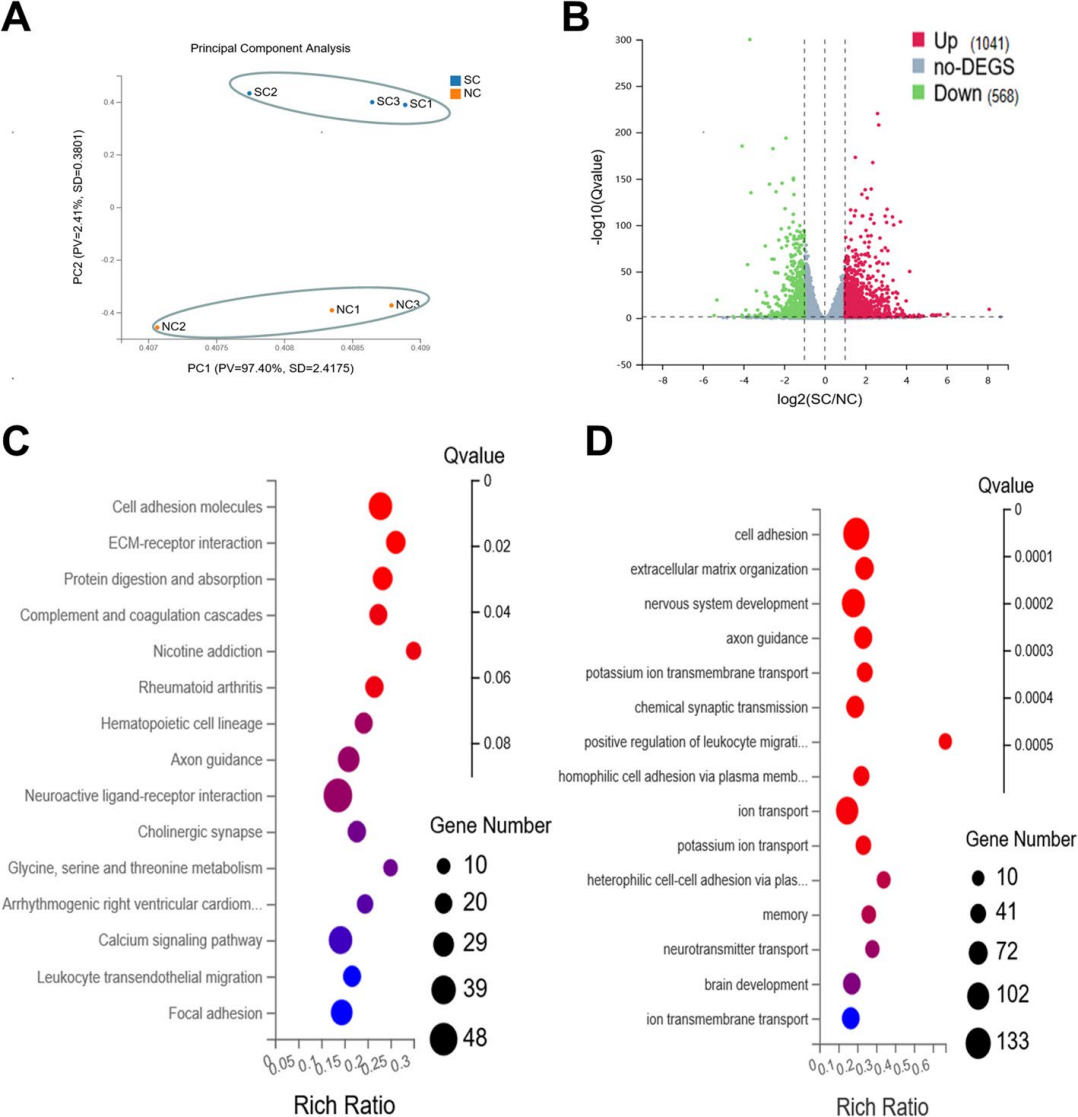
Transcriptomics analysis for the spheroid culture (SC) group. **A** Principal component analysis exhibited obvious differences between the two groups. **B** The volcano plot showed 1041 DEGs were up-regulated and 568 DEGs were down-regulated in the SC group. **C** The 15 significantly enriched signaling pathways based on DEGs. **D** The 15 significantly enriched Gene Ontology (GO) biological process (BP) terms based on DEGs

To further elucidate the mechanisms underlying the beneficial effects of hiPSC-RPE spheroids, we conducted bulk RNA sequencing analysis using the cells cultured with 3D spheroidal culture (SC) and 2D normal culture (NC) method. All cells were treated with Y-YR. The results of principal component analysis (PCA) showed obvious differences between the groups, with good reproducibility within each group (Fig. 6A). Subsequently, we set the screening threshold to |log2FoldChange|≥ 1 and Q-value ≤ 0.05 to identify differentially expressed genes (DEGs). As a result, 1041 genes were up-regulated and 568 genes were down-regulated, which was depicted in the volcano plot (Fig. 6B).

Next, Gene Ontology (GO) terms and the Kyoto Encyclopedia of Genes and Genomes (KEGG) pathway enrichment analysis were conducted to further analyze DEGs. The GO enrichment analysis identified 94 enriched GO terms associated with the biological process (GO-BP). Among these terms, the top 15 GO terms with the most significant differences are listed in the bubble chart, including cell adhesion, extracellular matrix (ECM) organization, nervous system development, axon guidance, potassium ion transmembrane transport, chemical synaptic transmission, positive regulation of leukocyte migration, homophilic cell adhesion via plasma membrane adhesion molecules, ion transport, potassium ion transport, heterophilic cell-cell adhesion via plasma membrane cell adhesion molecules, memory, neurotransmitter transport, brain development, and ion transmembrane transport (Fig. 6C). In the KEGG pathway analysis, 15 pathways were ranked by Q-value from low to high (Fig. 6D). Some of these pathways are meaningful to us, such as cell adhesion molecules and ECM-receptor interaction.

**Fig. 7:**
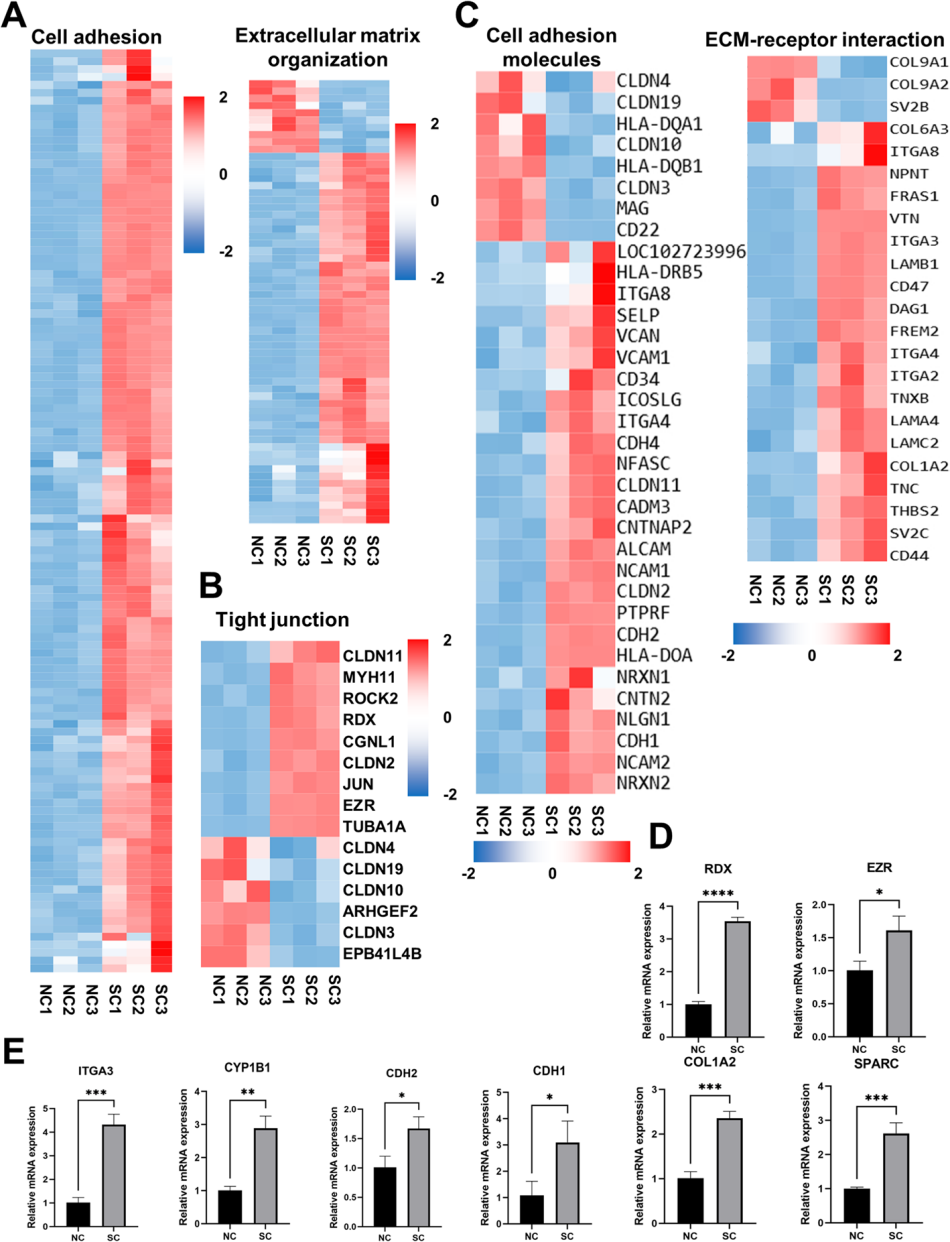
Heatmaps and gene validation of major GO-BP terms and KEGG pathways. **A** The heatmaps of cell adhesion and ECM organization in GO-BP analysis. **B**, **C** The heatmaps of tight junction, cell adhesion molecules and ECM-receptor interaction in KEGG enrichment analysis. **D**, **E** RT-qPCR results of mRNA expression for *CDH1, CDH2, CYP1B1, ITGA3, COL1A2, SPARC, RDX, EZR* in the NC and SC group. Mean ± SD (*p < 0.05; **p < 0.01; ***p < 0.001; ****p < 0.0001; *n* = 3)

In the following step, we selected the top two GO terms of GO-BP for further observation, including “cell adhesion” and “ECM organization”. The DEGs enriched in the two terms were presented as heatmaps (Fig. 7A). Furthermore, we selected meaningful pathways from KEGG enrichment results to analyze, including “cell adhesion molecules”, “ECM-receptor interaction”, and “tight junction”. Heatmaps were also drawn to visualize the differences between the two groups (Fig. 7B, C). Both the GO and KEGG analyses highlighted the presence of significant DEGs associated with ECM and cell adhesion in hiPSC-RPE spheroids. To validate the RNA-seq data, RT-qPCR was performed and the results demonstrated that the expression of cell adhesion-related genes (*CDH1, CDH2, CYP1B1, ITGA3*), ECM genes (*SPARC, COL1A2*), cell-junction related genes (*ITGA3, RDX, EZR, CDH1*) were significantly up-regulated in RPE cells with spheroid culture (Fig. 7D, E). These results were consistent with RNA-seq data.

### Tissue engineering RPE sheet derived from bioprinting hiPSC-RPE cell spheroids with supplementation of Y27632 and RepSox

**Fig. 8:**
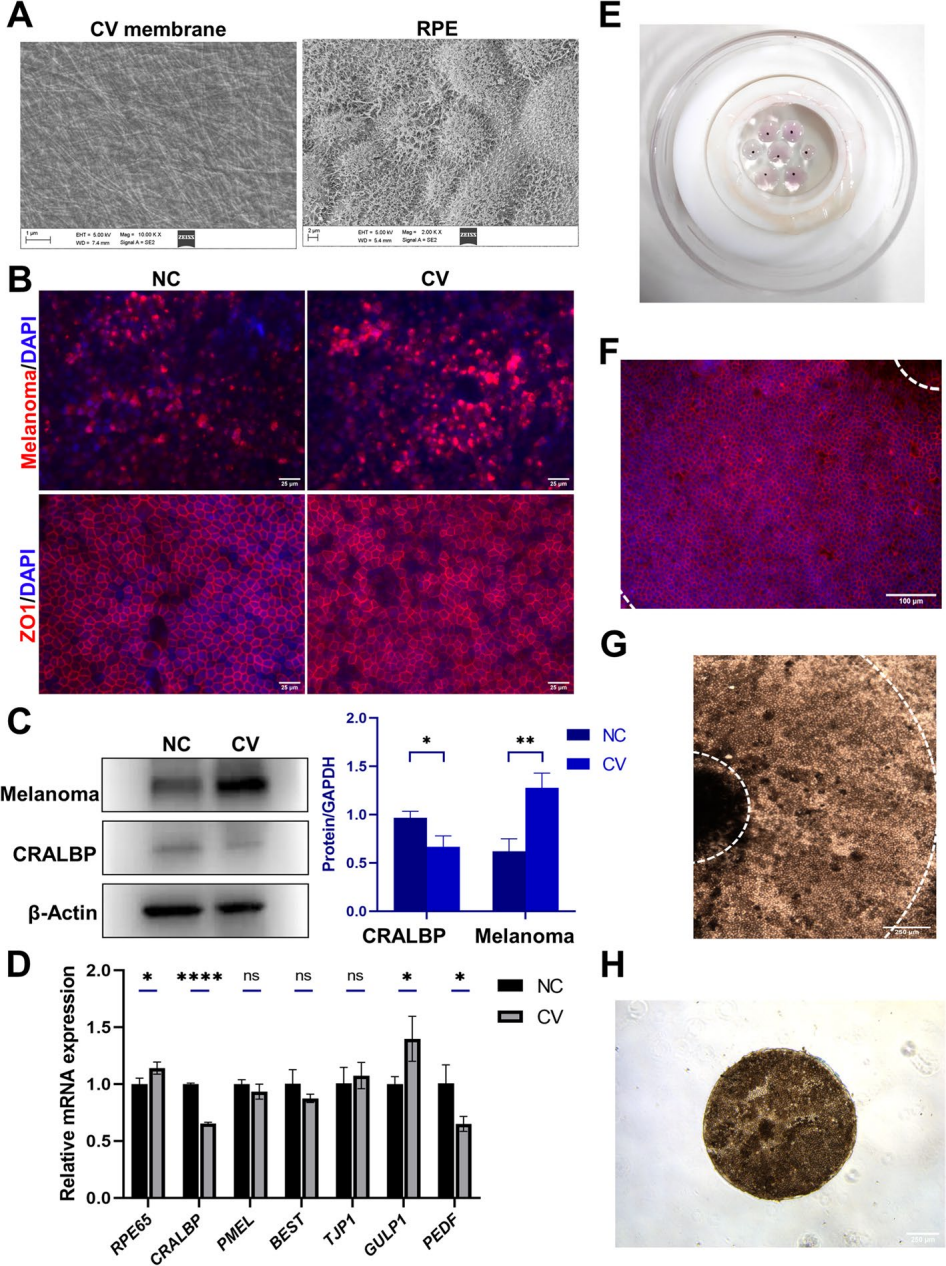
Engineered RPE patches on the collagen vitrigel (CV) membrane. **A** Scanning electron microscopy (SEM) of CV membrane and the RPE cells on CV membrane. Scale bar 1 μm. Scale bar 2 μm. **B** Immunofluorescence staining of ZO1 and Melanoma in the normal control (NC) group and CV group. Scale bar 25 μm. **C** Western blotting analysis of CRALBP and Melanoma expression in the two groups. **D** RT-qPCR results of mRNA expression for *RPE65, CRALBP, PMEL, BEST, TJP1, GULP1, PEDF* in the two groups. **E** The photograph of the bio-printed RPE spheroids. **F** The regular ZO1 expression of RPE in the circular region adjacent to spheroids. **G** The bright-filed image of the circular region adjacent to spheroids. **H** The collected RPE patches cutting from the engineered RPE sheets. Mean ± SD (*p < 0.05; **p < 0.01; ****p < 0.0001; *n* = 3)

After identifying the effects of Y-YR and spheroid culture on hiPSC-RPE cells, we next constructed RPE sheets using RPE spheroids and bioprinted them onto collagen vitrigel (CV) membrane which could be a scaffold for many types of cells [29–31]. Firstly, we seeded hiPSC-RPE spheroids onto collagen vitrigel (CV) membranes and tissue culture plates (TC plates) at a same density, supplemented with Y-YR. The images of scanning electron microscopy (SEM) showed that the CV membrane was characterized by an intricate network of crisscrossed collagen fibers and the RPE cells adjacent to hiPSC-RPE spheroids grew well with numerous microvilli on the CV membrane (Fig. 8A). On day 15, immunofluorescence staining was performed and the results showed that cells on both CV membranes and TC plates displayed good hexagonal shapes and ZO1 expression (Fig. 8B). The cells on CV membranes exhibited a significant increase in melanoma expression compared to the control group (Fig. 8B). Western blot results further confirmed an increase in melanoma protein expression and a concurrent reduction in CRALBP expression in cells cultured on the CV membrane (Fig. 8C). Quantification of western blot bands indicated significant differences in CRALBP expression (*P* < 0.05) and Melanoma expression (*P* < 0.01) (Fig. 8C). Next, we conducted RT-qPCR and the results demonstrated a significant down-regulation of *CRALBP* and *PEDF* RNA expression, coupled with a significant up-regulation of *RPE65* and *GULP1*(a gene related to phagocytosis) in the cells cultured on the CV membrane (Fig. 8D).

After that, we used a bioprinter to print RPE spheroids onto CV membranes. The pipettor of the bioprinter aspirated RPE spheroids and relocated them on CV membranes which were gripped by polytetrafluoroethylene holder (Fig. S4A) and were loaded on a mobile platform of bioprinter. The platform can move along the X-axis or the Y-axis following a preset print pattern (Fig. S4B); therefore the RPE spheroids could be relocated as we expected (Movie S1). After repeated commissioning, we successfully printed RPE spheroids following the preset pattern and the RPE spheroids could be evenly distributed through the bioprinting (Fig. 8E). The spheroids without bioprinting easily aggregated together on CV membranes and the expansion of spheroids were limited (Fig. S5A). While the bioprinted spheroids could be dispersed evenly, and then expand and grow well (Fig. S5B, C). Additionally, we found the hiPSC-RPE cells adjacent to spheroids (approximately 1.1 mm circular band) showed a homogeneous tight junction (Fig. 8F). Building on this observation, we employed a 1 mm trephine to precisely cut and collected the RPE patches situated in such circular band. The specific cutting area is within the region marked by white dashed circles in Fig. 8G. This technique enabled us to successfully construct RPE patches characterized by the presence of more mature hiPSC-RPE cells (Fig. 8H).

## Discussion

Many differentiation protocols have been successfully developed for generating RPE from hiPSCs and hESCs [27, 32, 33]. However, in these established protocols, the process of obtaining mature and qualified RPE cells for cell therapy typically takes several months, making it a time-consuming and somewhat inefficient endeavor. Therefore, achieving rapid access to high-quality RPE cells remains to be a challenge. In this study, we illustrated that the supplement of Y27632 and Repsox could help obtain qualified hiPSC-RPE cells. Moreover, we demonstrated the superior adhesion and ECM organization achieved through RPE spheroid culture compared to conventional 2D culture methods. After that, we combined RPE spheroids with the supplementation of Y27632 and Repsox, facilitating the rapid production of mature RPE cells. We further utilized these cells to construct engineered hiPSC-RPE sheets on CV membranes, employing both seeding and bioprinting techniques. At last, we harvested high-quality hiPSC-derived RPE patches which could be used for cell therapy in future.

ROCKs play a pivotal role in regulating various cellular functions in non-muscle cells by controlling actin-cytoskeleton assembly, influencing processes like stress-fiber formation and cell adhesion [34]. In the context of hiPSC-RPE, the inhibitors of ROCKs contribute to adhesion, extended passages, anti-apoptosis, pigmentation, and morphological characteristics of iPS-RPE [13, 14]. However, it is worth noting that ROCK inhibitors have also been reported to increase permeability in epithelial cells and impair cell polarization, resulting in the formation of random protrusions and multiple leading edges in certain cell types [34, 35]. Our study aligns with previous research by confirming that Y27632, as a ROCK inhibitor, can indeed enhance cell adhesion and viability of hiPSC-RPE cells while inducing multiple protrusions in these cells through the reorganization of F-actin. Subsequently, we introduced Repsox, an effective TGF-β signaling inhibitor, to act on hiPSC-RPE cells with Y27632. The TGF-β signaling pathway is known to regulate many biological functions including cell proliferation, differentiation, apoptosis, adhesion, and migration through the Smad and non-Smad pathways [36]. The TGF-β signaling pathway also promotes EMT and the inhibition of it can reduce the passage-dependent loss of epithelial potential [37]. Based on its effect on the TGF-β signaling pathway, Repsox is used in cellular reprogramming and EMT intervention [18, 37]. It has also been reported to help maintain epithelial-like morphology in primary mouse RPE cells [18, 38]. In our experiments, we found the addition of Repsox led to decreased cell viability and increased apoptosis of hiPSC-RPE during seeding, consistent with the observations of He D et al., who noted RepSox-induced apoptosis in osteosarcoma cells [36]. Therefore, our results suggest that Repsox may not be conducive to long-term RPE culture. In contrast, the combined use of Y27632 and Repsox contributed to the enhanced cell adhesion of hiPSC-RPE cells, though the effects are mainly due to Y27632.

We then conducted a 2-week study to assess the effects of RepSox, Y27632, and Y-YR on hiPSC-RPE cells. Our results revealed that a 2-week supplement of Y-YR in cells in 2D conditions caused a significant up-regulation in the expression of *NCAD* (also known as *CDH2*), *ECAD* (also known as *CDH1*), and *CRALBP*. While in RPE spheroids, the expression of *CRALBP* is not up-regulated significantly. *CRALBP* is a well-established RPE-related gene that plays a crucial role in the visual cycle by stimulating the enzymatic isomerization of all-trans-retinol to 11-cis-retinol [39]. The discrepancy might be due to the 3D spheroid culture environment in which cells are under different chemical milieu and physical forces [40]. Our previous study confirmed that the stiffer substrate promoted cell differentiation and softer substrate was more effective in maintaining the phenotype of cells [41]. Therefore, compared to 2D culture (cell-TCP contact), we consider that the 3D spheroid environment (cell-cell contact) might provide a softer physical environment to help in maintaining phenotypes when external environment changed. Compared to the control group and Y27632 group, the up-regulation of *NCAD* and *ECAD* in Y-YR group was significantly. Both ECAD (also known as E-cadherin) and NCAD (also known as N-cadherin) are pivotal proteins in the superfamily of calcium-dependent intercellular adhesion molecules. Though N-cadherin is implicated in promoting mesenchymal phenotype in carcinoma cells and EMT, it is abundantly expressed in RPE cultures [42]. E-cadherin is also expressed in RPE cells and it is reported to play an important role in maintaining epithelial cell phenotypes. In RPE cells, the overexpression of E-cadherin could inhibit the EMT process and cell proliferation [43]. Based on the above consideration, we believe that the addition of Y27632 at an early stage of hiPSC-RPE culture and the short-term supplementation of Y27632 + Repsox at a later stage of hiPSC-RPE culture can promote the epithelial phenotype of RPE cells.

As we observed, the dispersed hiPSC-RPE cells in agarose micro-molds had the propensity to organize into aggregates or spheroids, which is intricately linked to the cortical cytoskeleton, cell-cell adhesions, and cell-matrix adhesions [44]. When we constructed RPE spheroids, we found that both Y27632 and Y27632 + Repsox significantly promoted the spheroid formation and the overall survival of RPE spheroids. Considering that Y27632 is known to regulate the cytoskeletal system [45], we hypothesized that it might reorganize the cellular stress fiber to facilitate the formation of robust RPE spheroids. The effects of YR are mainly due to Y27632. Throughout the extended cultivation of RPE spheroids, we found that both Y27632 and Y-YR treatments significantly promoted the expansion of RPE spheroids. In contrast, spheroids without any additive exhibited poor adhesion and limited expansion. It is well-known that cells situated deep inside spheroids often face challenges related to inadequate nutrient and oxygen supply [46]. Consequently, the central region of a cell spheroid tends to undergo apoptosis and necrosis due to limitations in mass transport [47, 48]. Thus, only the RPE cells that expanded from the spheroid core could persist. In light of this, our study underscores the critical role of Y-YR treatment in supporting the growth of RPE cells in spheroids.

Spheroid cultures are widely used for their capacity to mimic a 3D physiological environment [49], providing cells with enhanced cell-cell and cell-ECM interactions [33]. In the current study, we conducted RNA sequencing to determine the key disparities between spheroid and conventional 2D cultures. Building upon our GO-BP analysis findings, we focused on two key aspects: cell adhesion and ECM organization. *CDH2*, *ITGA3*, *COL1A2*, and *CYP1B1* were selected and validated to be up-regulated in RPE spheroids. *CDH1*, *SPARC*, *COL1A2*, and *ITGA3* were selected from the ECM organization and were also up-regulated in RPE spheroids. *CDH1* and *CDH2* are both important molecules for cell adhesion. Membrane-bound CDH2, which is predominant in RPE cells, is of fundamental importance for cell adhesion in RPE [42]. CYP1B1, as a member of the cytochrome P450 superfamily, is involved in the metabolism of fatty acid, retinoic acid, and 17b estradiol [50]. It has also been implicated in the regulation of cell adhesion and migration [51]. *CYP1B1* is a classic target gene of the aryl hydrocarbon receptor (AhR), which can be activated by switching cells from adherent to suspension culture [52]. This suggests that the spheroid culture of hiPSC-RPE cells can initially trigger AhR recruitment, leading to long-term up-regulation of *CYP1B1*, which could, in turn, be involved in cell adhesion and retinoic acid metabolism. ITGA3 is a member of the integrin family. It can bind to extracellular ligands present in Bruch’s membrane and promote the adhesion of RPE cells [53]. While the expression of these adhesion molecules is crucial, these molecules must connect with ECM to be adhesive. In our research, we found that ECM-related genes including *SPARC* and *COL1A2* were also up-regulated. SPARC (Secreted Protein, Acidic and Rich in Cysteine) is synthesized and secreted by RPE and it plays an important role in matrix assembly and cell-matrix interactions in RPE cells [54]. The decline of SPARC protein may contribute to the pathogenesis of AMD [54]. Thus, our results reveal that spheroid culture can enhance cell adhesion and ECM organization by up-regulating both adhesion and ECM-related genes. KEGG enrichment analysis also indicated up-regulation of genes related to cell adhesion molecule and ECM-receptor interaction. Furthermore, we also found some tight junction-related genes (*EZR*, *RDX*) were up-regulated in the spheroid culture group.

Assessing of the maturation status of RPE cultures involves evaluating tissue-specific protein expression, protein polarity, and pigmentation [42]. In our study, we found the hiPSC-RPE cells located close to spheroids exhibited higher expression of BEST and melanoma. Moreover, the cells in this region displayed lower levels of Ki67, a marker of proliferation. Bestrophin is an integral membrane protein localized to the basolateral plasma membrane [55]. As RPE cells mature, the expression of BEST typically increases [56]. Therefore, we consider the RPE cells derived from spheroids and situated adjacent to spheroids to be a valuable cell source due to their enhanced maturity and reduced proliferative capacity. Subsequently, we constructed a scaffold using CV membrane. CV membrane has been reported to serve as a scaffold for limbal epithelial cells and supports corneal epithelial differentiation while preventing epithelial hypertrophy [30]. Additionally, collagen vitrigel is reported to be a structurally and mechanically robust substrata for hESC-RPE culture [57]. They also reported that the gene expression of hESC-RPE varied on TCP and collagen vitrigel after 4 and 6 weeks culture [57]. In the current study, we found some specific RPE markers were up-regulated in the cells cultured on CV membranes after a 2-week culture, though the expression of *CRALBP* decreased. Additionally, as we mentioned above, substrate stiffness has impact on cell phenotypes [41]. The down-regulation of *CRALBP* might be affected by the softer CV membrane and a longer culture time should be considered when cultivating RPE cells on CV membrane in future studies.

Compared to RPE cell suspension, RPE patches can be easily delivered to a target area in retina and the cells on patches are in a form close to their native configuration with tight junction formed [7, 11]. Several literature reported RPE sheets used for clinical transplantation. Mandai et al. used 1.3 × 3 mm hiPSC-RPE sheets without artificial scaffold for transplantion and found the sheets were curled after operation [5]. Other kind of RPE sheets are reported in size of 3.5 × 6.25 mm and 6 × 3 mm with ~ 100,000 cells on sheets and both studies used synthetic substrates (parylene substrate and polyethylene terephthalate parylene substrate respectively) [6, 7]. Compared to these studies, we adopted a natural biomaterial of CV membrane, which is well-tolerated in vivo [30]. Then, we selected and cut the circular RPE patch in size of 1 mm diameter with approximately 4000–5000 RPE cells on it. It is known that the fovea is of the highest visual acuity in human eyes and most of vision loss diseases are related to the dysfunctional cells of this area. Therefore, RPE cell delivery should focus on this area. Accordingly, the diameter of fovea and macula is about 0.8–1.5 mm and 5.5 mm respectively [58]. Though our patches are in small size, they might be delivered to fovea and macular area with multipoint injection according to the area of atrophic RPE in future treatment.

In the current study, the RPE cells expressed relatively homogeneous tight junction in the region adjacent to RPE spheroids within 1.1 mm. Moreover, the BEST expression in this region is relatively uniform too, compared to the cells cultured in 2D conditions. Considering that the pigmentation of RPE cells requires a long culture time. We think a longer culture time might be needed to get a more uniform pigment expression in RPE spheroids in future studies. Therefore, in the future, we will improve the technique of spheroid bioprinting and suitable culture conditions with a longer culture time to obtain a larger area of RPE patch with a more uniform confluent RPE distribution.

## Conclusions

Our study developed an engineered hiPSC-RPE patch with improved viability and characteristics, as well as reduced proliferative capacity using RPE spheroids, Y-YR supplementation and the CV scaffold. These developed RPE patches present a promising and practical approach for developing clinical-grade hiPSC-RPE sheets and RPE cell therapy. However,our study has some limitations. First, we did not conduct transplantation experiments to assess the practicality of our RPE patches in vivo, necessitating further investigation with delicate tissue manipulation and suitable surgical micro-instruments. Due to the small size of RPE patches, subretinal injection may be a potential delivery mode. Second, we did not explore the potential of using RPE spheroids and engineered RPE sheets to construct a retinal co-culture system, which could serve as an in vitro retina model. Future experiments can focus on bioprinting techniques for constructing engineered cell sheets and developing co-culture methods to combine RPE sheets with retinal organoids or retinal explants.

## Materials and methods

### hiPSCs culture and RPE cell differentiation

The normal hiPSC cell line was cultured on Matrigel-coated plates(Corning, USA) using hiPSC medium (NuwacellTM hiPSC/hESC medium ncTarget; Nuwacell, Anhui, China). The hiPSCs were dissociated with 0.5 mM EDTA(Nuwacell) at 80% of confluence and passaged every 3–4 days. The culture medium was changed daily.

The hiPSC-RPE cells were differentiated as previously reported [27]. Briefly, hiPSCs were cultured in a differentiation medium composed of Dulbecco’s modified Eagle’s medium (DMEM, high glucose, Gibco,Thermo Fisher Scientific, Waltham, MA, USA), supplemented with 50 μM β-mercaptoethanol (Sigma-Aldrich Corp., St. Louis, MO, USA), 1 × minimum essential medium–nonessential amino acids (MEM NEAA; Gibco), 1% penicillin-streptomycin (Gibco), and 20% of knockout serum replacement (KSR; Gibco) from D0-D42. 10 mM Nicotinamide (Sigma) was added to the differentiation medium at D0-D7, and 100 ng/ml Activin A (Peprotech, Thermo Fisher Scientific) and 3 µM CHIR99021 (Med Chem Express, New Jersey, USA) were added at D7-D14, D14-D42 respectively. On day 42, the pigmented cells were dissociated by TrypLE Express Reagent (Thermo Fisher Scientific) and enriched. Cells were then seeded on Matrigel-coated culture plates at a density of 3.5 × 104/cm^2^ in a maintained medium composed of high glucose DMEM, 4% KSR, 1 × MEM NEAA, 1% penicillin-streptomycin and 50 μM β-mercaptoethanol. The culture medium was changed every 2–3 days.

### Generation and seeding of RPE spheroids

RPE cells were dissociated as described above. The collected hiPSC-RPE cells were seeded onto specific agarose micromolds as described previously [23] and incubated for 48 h. During this time, the suspended cells formed into a spheroid-like aggregate. After incubation, the RPE spheroids were seeded or bioprinted for further use.

### Cell counting kit-8 assay (CCK-8)

A cell counting kit-8 (Bimake, Houston, USA) assay was performed to evaluate the viability of hiPSC-RPE cells. Briefly, RPE cells were seeded in a 96-well plate at a same density for 24 h. Then, Y27632 (10 μM) (Med Chem Express), Repsox (10 μM) (Med Chem Express) and a combination of Y27632 (10 μM) + Repsox (10 μM) were added into the culture medium, and the cells were further for additional 24 h. Then, 10 μL CCK-8 reagent was directly added into each well and the plate was incubated for 2 h until the color of medium turned orange. Subsequently, the absorbance value of the medium was read at 450 nm with a microplate reader.

### Live/dead cell assay

The viability of RPE spheroids was evaluated using a live/dead cell imaging kit (Invitrogen, Thermo Fisher Scientific) following the manufacturer’s instructions. RPE spheroids were exposed to a 1 × Live/dead working solution for 25 min at 37℃ in the dark. RPE spheroids were imaged using a fluorescence microscope (Olympus, Japan). The percentage of live cells was acquired from three independent samples.

### Relative quantification of pigmented area

To compare the expansion ability of RPE spheroids, the relative quantification process of the pigmented area was conducted with imageJ. The original color images of three groups were first combined into one image. Then, the combined images were converted to 8-bit grayscale images followed by the find edge process, threshold adjustment, target area setting, and the measurement of mean gray value (Fig. S6).

### Flow cytometry

For the cell apoptosis experiment, hiPSC-RPE cells were dissociated, and the RPE suspension was divided evenly and treated with Y27632 (10 μM), Repsox (10 μM) and Y27632 (10 μM) + Repsox (10 μM), respectively. The Annexin V-Alexa Fluor 488/PI Kit (4A biotech, Beijing, China) was used. First, the cell suspension was centrifuged, washed with PBS, then centrifuged again. Second, the RPE cells were re-suspended in 1 × binding buffer and incubated with annexin V-Alexa Fluor 488 for 5 min. Third, propidium iodide solution was added to the cell samples, and the samples were detected immediately and analyzed using a flow cytometer (Novocyte).

### Immunocytochemistry

Cells were fixed in 4% paraformaldehyde for 15 min at room temperature and rinsed thrice with PBS. Following that, the cells were permeabilized in 0.1% Triton X-100 in PBS for 10 min and blocked with 3% BSA in PBS for 1 h at room temperature. Next, the cells were incubated with primary antibodies overnight at 4℃. After rinsing with PBS, the cells were stained with Alexa Fluor-conjugated secondary antibodies (Thermo Fisher Scientific, 1:1000) for 1 h at room temperature. Following PBS wash, the cells were stained with DAPI for 5 min and mounted with a mounting medium. Immunofluorescence was examined using a fluorescence microscope (Olympus, Japan). CoraLite594-Phalloidin (Proteintech, USA) conjugates were used to label filamentous actin (F-actin). The other antibodies used are listed in Table S1.

### Reverse Transcription and Quantitative Real-time PCR (RT-qPCR)

Total RNA was extracted using the FastPure® Cell Total RNA Isolation Kit (Vazyme, Nanjing, China) and dissolved in RNase-free water. RNA samples were quantified by measuring the OD value at 260 nm, and the OD 260/280 ratios for all RNA samples fell within the range of 1.8 to 2.1. Reverse transcription was performed using the HiScript III RT SuperMix for qPCR (+gDNA wiper) kit (Vazyme), following the manufacturer’s instructions. RT-qPCR was conducted using the ChamQ Universal SYBR qPCR Master Mix kit (Vazyme), and the PCR mixture was run in the CFX96 Real-Time PCR system (Bio-Rad, USA). Three technical replicates were set and expression levels were normalized to the expression of GAPDH. Relative expression levels compared to the control group were determined by calculating the 2 - ^ΔΔCt^.

### Western blotting

The cells were washed twice with PBS, then treated with RIPA buffer (Thermo Fisher Scientific), and centrifuged at 12,000 rpm for 15 min. The supernatant was reserved. The protein concentration of the samples was determined using a BCA protein assay kit (Thermo Fisher Scientific). The protein samples were adjusted to a concentration of 2 μg/μl. Subsequently, 10 μl of the protein samples were loaded and separated by electrophoresis on a 10% SDS-PAGE gel (SDS-PAGE gel kit, Solarbio Life Sciences, Beijing, China). Following protein separation, the proteins were transferred to PVDF membranes (Millipore, Billerica, MA, USA), blocked with 5% BSA at room temperature for 1 h, and then incubated with primary antibodies overnight at 4℃. The membrane was washed three times with TBST and subsequently incubated with HRP-conjugated secondary antibodies for 2 h at room temperature. After further washing with TBST, protein bands were visualized using an ECL kit (Millipore, USA) with a gel imager. The antibodies used are listed in Additional file 3, Table S1.

### Karyotype analysis

The karyotype analysis were conducted using a standard G-band technique (350G–400G) and analyzed by Ikaros karyotyping system. 20 metaphases were examined. The number of chromosomes as well as the presence of structural chromosomal abnormalities was examined.

### Transepithelial electrical resistance (TER) assay

TER assay was conducted using the EVOM2 voltohmmeter (World Precision Instruments, Sarasota, Florida, USA), following the manufacturer’s instructions. RPE cells and RPE spheroids were seeded onto PET Transwell membranes (3 μm pore size, Corning) coated with Matrigel at a same density and cultured for 2 weeks. TER values were measured from three replicates on day 14. Background resistance was determined from a blank culture insert. The TER value (Ωcm^2^) was calculated using the following formula.

$$\mathrm{TER }(\Omega /{{\text{cm}}}^{2}) = ({{\text{R}}}_{{\text{total}}}-{{\text{R}}}_{{\text{insert}}})/{\text{A}}$$

R_total_ represents the total resistance measured (Ω), R_insert_ represents the resistance of the blank insert, and A represents the membrane area (cm^2^) of the insert.

### Phagocytosis assay

POS was obtained from fresh porcine eyes, isolated in sucrose gradient solution, and centrifugated at 106,000 × g for 50 min at 4 ℃, following established protocols [59, 60]. Then, POS was labeled with FITC isomer 1 (0.5 mg/ml) in DMEM for 1 h at room temperature in the dark. RPE cells were exposed to the labeled POS solution containing 10%FBS for 6 h at 37℃. After incubation, RPE cells were thoroughly washed with PBS, fixed with 4% paraformaldehyde, permeabilized with 0.1% Triton X-100, and blocked with 3% BSA. Subsequently, the ZO-1 antibody was used for immunolabelling, and nuclei were stained with DAPI. Imaging was performed using a confocal microscope (Leica TCS SP8, Germany).

### RNA-sequencing analysis

Total RNA was isolated from hiPSC-RPE in 2D culture and spheroid culture way with Trizol total RNA reagent (Invitrogen). RNA sequencing was performed by the DNBSEQ platform (BGI Tech, Shenzhen,China). After the filtration of raw data, the clean reads were generated and aligned to the human genome assembly. After that, the qualified reads were standardized and quantified with transcripts per kilobase million(TPM) values. Differentially expressed genes(DEGs) were identified between the two groups. The significance of DEGs was defined by the combination of |log2FoldChange|≥ 1 and Q-value ≤ 0.05. Gene Ontology (GO) terms enrichment and the Kyoto Encyclopedia of Genes and Genomes (KEGG) enrichment analysis were performed to further analyze data.

### Fabrication of collagen vitrigel (CV) membrane

The CV membrane was constructed as previously described [61]. Briefly, 5 mg/ml pre-cooling type I collagen solution (extracted from bovine tendon, Guangzhou Trauer Biotechnology, China) and 10 × DMEM (Hyclone, USA) were mixed in volume of 1:9. The mixed collagen hydrogel was centrifugated (2000 rpm, 3 min) and added into 6-well plates. The plates were then incubated at 37℃ to complete the gelation of the collagen for 30 min and dried in a clean air chamber at 10℃ and 40% humidity for 8 h to roughly remove the moisture of the gel. After that, the collagen gel was dried on a clean table and exposed to ultraviolet rays at room temperature for additional days to achieve vitrification. Then, the vitrified membrane was rehydrated with PBS. Using tweezers, the CV membrane was gently removed and placed into a ring-shaped polytetrafluoroethylene mold for the subsequent loading of RPE cells.

### Scanning electron microscope (SEM)

SEM was used to examine the ultrastructural surface of the CV membrane and the RPE cells cultured on the CV membranes. RPE spheroids were initially seeded onto the CV membrane and cultured for 14 days. Subsequently, the cell sample and CV membrane were fixed in 2.5% glutaraldehyde for 2 h, followed by three washes with a PBS solution. The cell samples were then dehydrated using a series of increasing ethanol concentrations in PBS (50%, 70%, 80%, 90%, 95%, 100%, 100%). Afterward, the samples underwent critical point drying and gold metal coating, and were imaged using a scanning electron microscope (Zeiss, Ultra 55).

### Automatic RPE spheroids bioprinting

The automatic spheroids bioprinting device was designed and assembled by our team. Its precision and accuracy were validated through calibration and testing conducted by a certified center (Ceprei, China; Certificate NO. 1GA18004742-0002). RPE spheroids from 96-well U-shaped plates were aspirated and accurately printed onto the surface of CV membranes automatically following a preset pattern (Movie S1). Subsequently, culture medium was gently added to the cell plates, and they were placed in an incubator at 37℃ with 5% CO_2_ for further cultivation.

### Statistical analysis

The data are presented as means ± standard deviation (SD) and derived from a minimum of three samples. GraphPad Prism 6.0 (GraphPad Software, San Diego, CA, USA) and ImageJ (National Institutes of Health, Bethesda, MD, USA) were employed for data analysis. The unpaired two-tailed t-test was applied for comparison between groups. *P* values < 0.05 were considered statistically significant.

### Supplementary Information

**Additional file 1: Figure S1.** Quantification of MITF positive cells. **Figure S2.** The results of karyotype analysis. **Figure S3.** Formation of RPE spheroids in agarose molds. (A) The photograph of agarose molds. (B, C) Bright-field images of the RPE spheroids formed in agarose molds. Scale bar 250μm. Scale bar 50μm. **Figure S4.** The process of RPE spheroids bioprinting. (A) The photographs of the CV membrane. (B) The preset print pattern of bioprinting. (C) The photograph of the bioprinter. **Figure S5.** The bioprinted RPE spheroids on CV membranes. (A) The photograph of RPE spheroids without bioprinting. (B) The bioprinted RPE spheroids. (C) The expansion of bioprinted RPE spheroids. **Figure S6.** Process for quantification of the pigmented area.

**Additional file 2: Movie S1.** Bioprinting RPE spheroids on CV scaffolds.

**Additional file 3: Table S1.** List of primary antibodies. **Table S2.** The top 15 KEGG signaling pathways. **Table S3.** The top 15 GO biological process terms.

## Data Availability

The datasets used and/or analysed during the current study are available from the corresponding author on reasonable request.

## Abbreviations

RPE: Retinal pigment epithelium
BRB: Blood-retinal barrier
hiPSC: Human induced pluripotent stem cell
ECM: Extracellular matrix
CV: Collagen vitrigel
POS: Photoreceptor outer segments
3D: Three dimensional
hESCs: Human embryonic stem cells
TER: Transepithelial electrical resistance
ROCK: Rho-associated protein kinase
TGF-β: Transforming growth factor-beta
EMT: Epithelial-mesenchymal transition
TC: Tissue culture
SEM: Scanning electron microscope
