# Supplementary material for: Tissue engineering RPE sheet derived from hiPSC-RPE cell spheroids supplemented with Y-27632 and RepSox
Source: J Biol Eng. 2024 Jan 16;18:7. doi: 10.1186/s13036-024-00405-8 (PMC10790375; doi:10.1186/s13036-024-00405-8)
Supplement: Supplementary file 1 — Additional file 1: Figure S1. Quantification of MITF positive cells. Figure S2. The results of karyotype analysis. Figure S3. Formation of RPE spheroids in agarose molds. (A) The photograph of agarose molds. (B, C) Bright-field images of the RPE spheroids formed in agarose molds. Scale bar 250μm. Scale bar 50μm. Figure S4. The process of RPE spheroids bioprinting. (A) The photographs of the CV membrane. (B) The preset print pattern of bioprinting. (C) The photograph of the bioprinter. Figure S5. The bioprinted RPE spheroids on CV membranes. (A) The photograph of RPE spheroids without bioprinting. (B) The bioprinted RPE spheroids. (C) The expansion of bioprinted RPE spheroids. Figure S6. Process for quantification of the pigmented area. [file 13036_2024_405_MOESM1_ESM.doc]

**
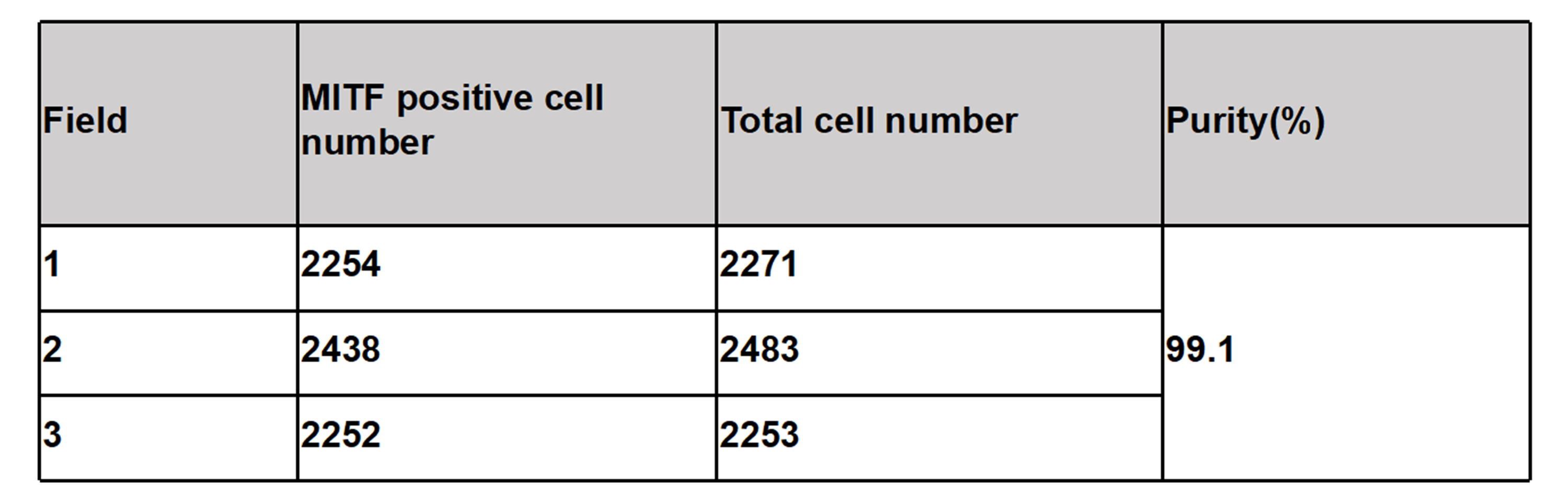
Figure S1.** Quantification of MITF positive cells. The purity of hiPSC-RPE cells was quantified using immunostaining for MITF. Purity was calculated by dividing the number of MITF positive cells by total cell number.


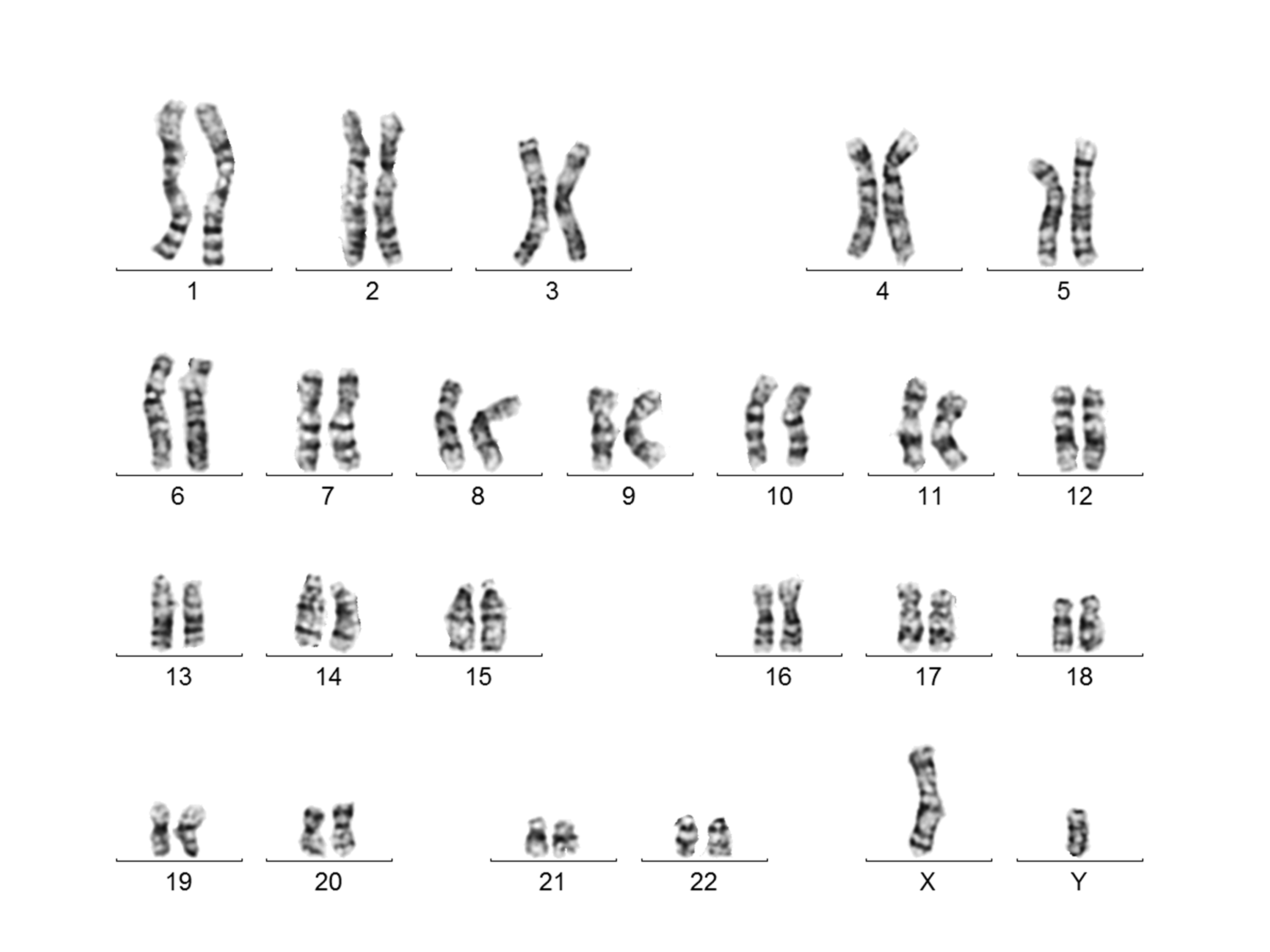


**Figure S2.** The results of karyotype analysis. hiPSC-RPE cells were treated with Y-YR for 14days and harvested for karyotype analysis using a G-banding technique. The cells were karyotypically normal 46, XY.

**
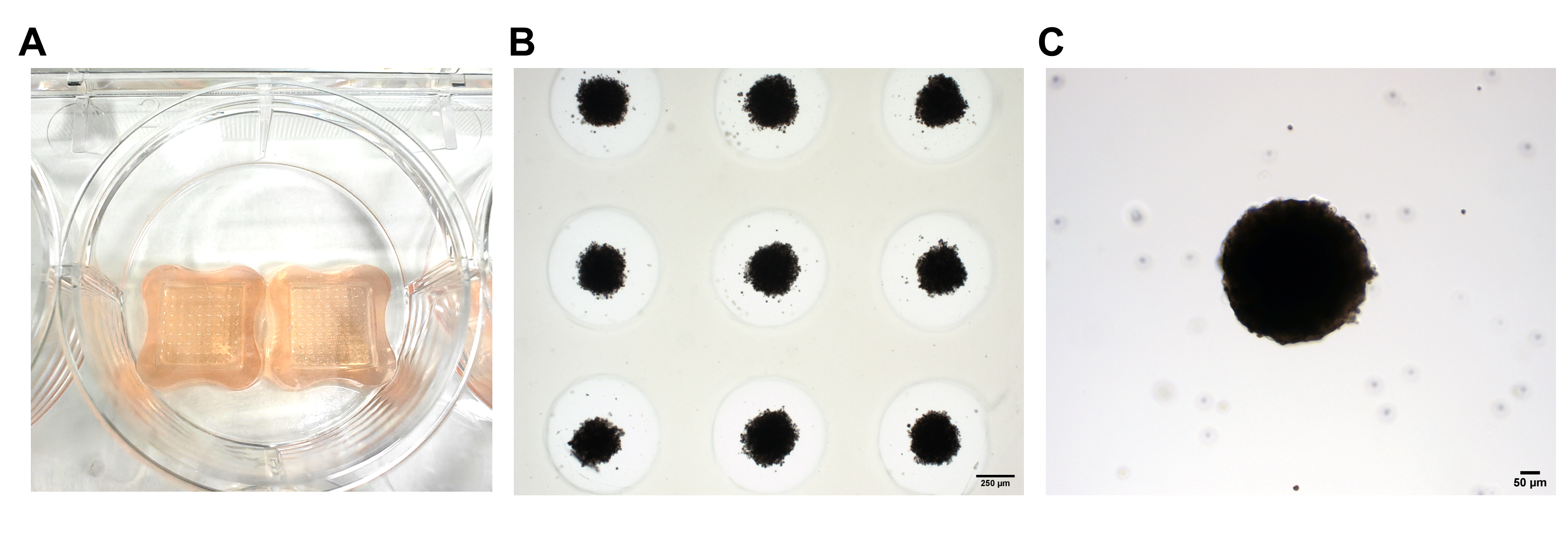
**

**Figure S3.** Formation of RPE spheroids in agarose molds. **(A)** The photograph of agarose molds. **(B, C)** Brightfield images of the RPE spheroids formed in agarose molds. Scale bar 250μm. Scale bar 50μm.

**
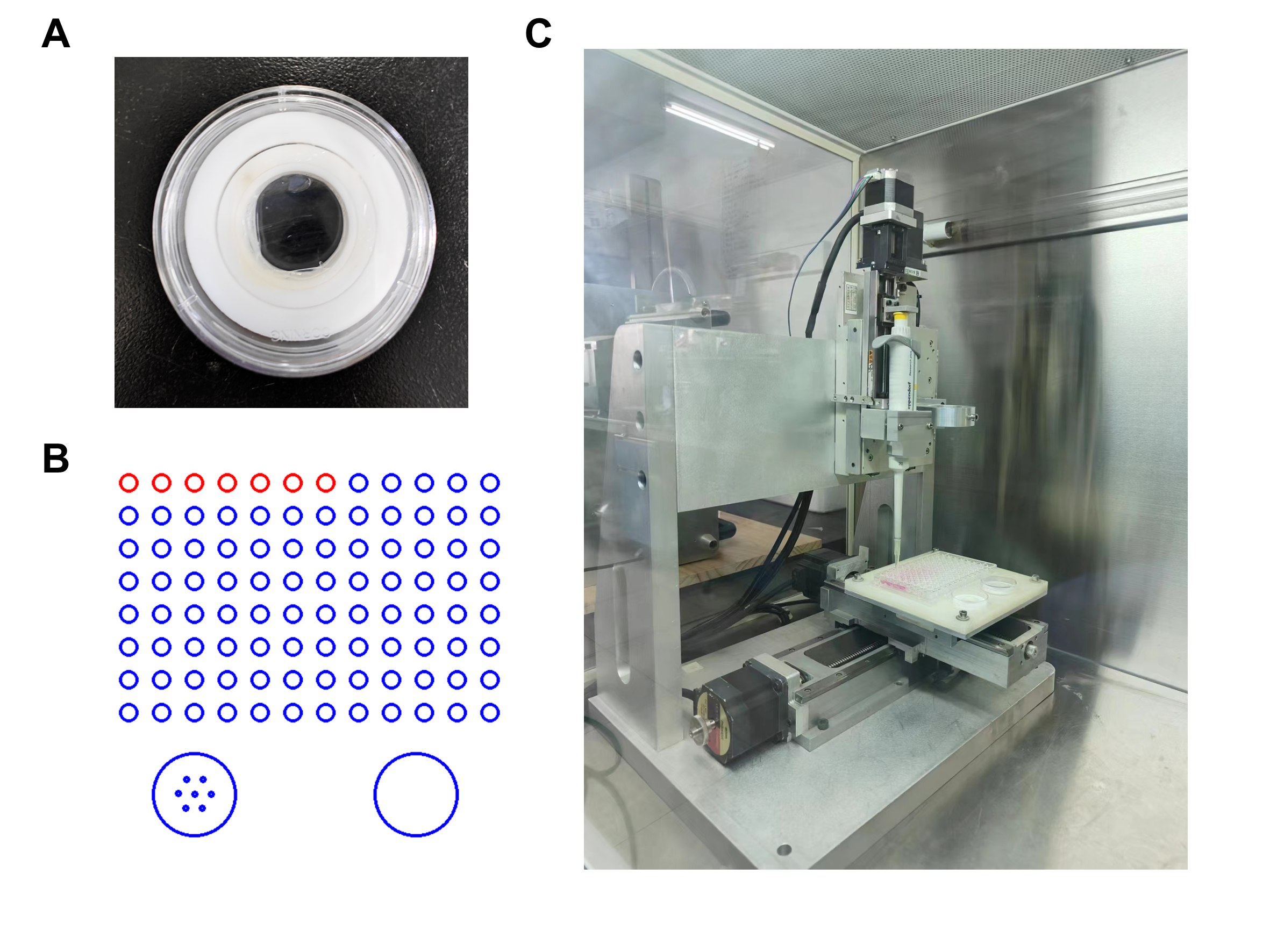
**

**Figure S4.** The process of RPE spheroids bioprinting. **(A)** The photographs of the CV membrane. **(B)** The preset print pattern of bioprinting. **(C)** The photograph of the bioprinter.


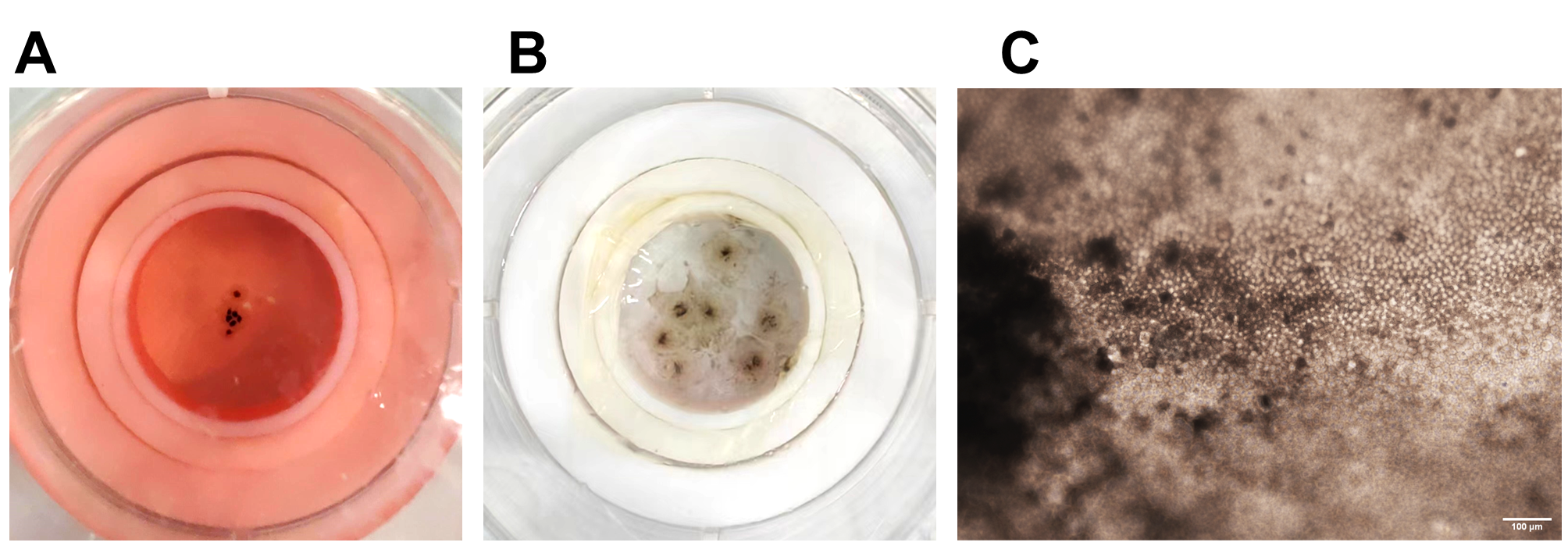
**Figure S5.** The bioprinted RPE spheroids. **(A)** The photograph of RPE spheroids without bioprinting. **(B)** The bioprinted RPE spheroids. **(C)** The expansion of bioprinted RPE spheroids.

**Figure S6.** Process for quantification of the pigmented area
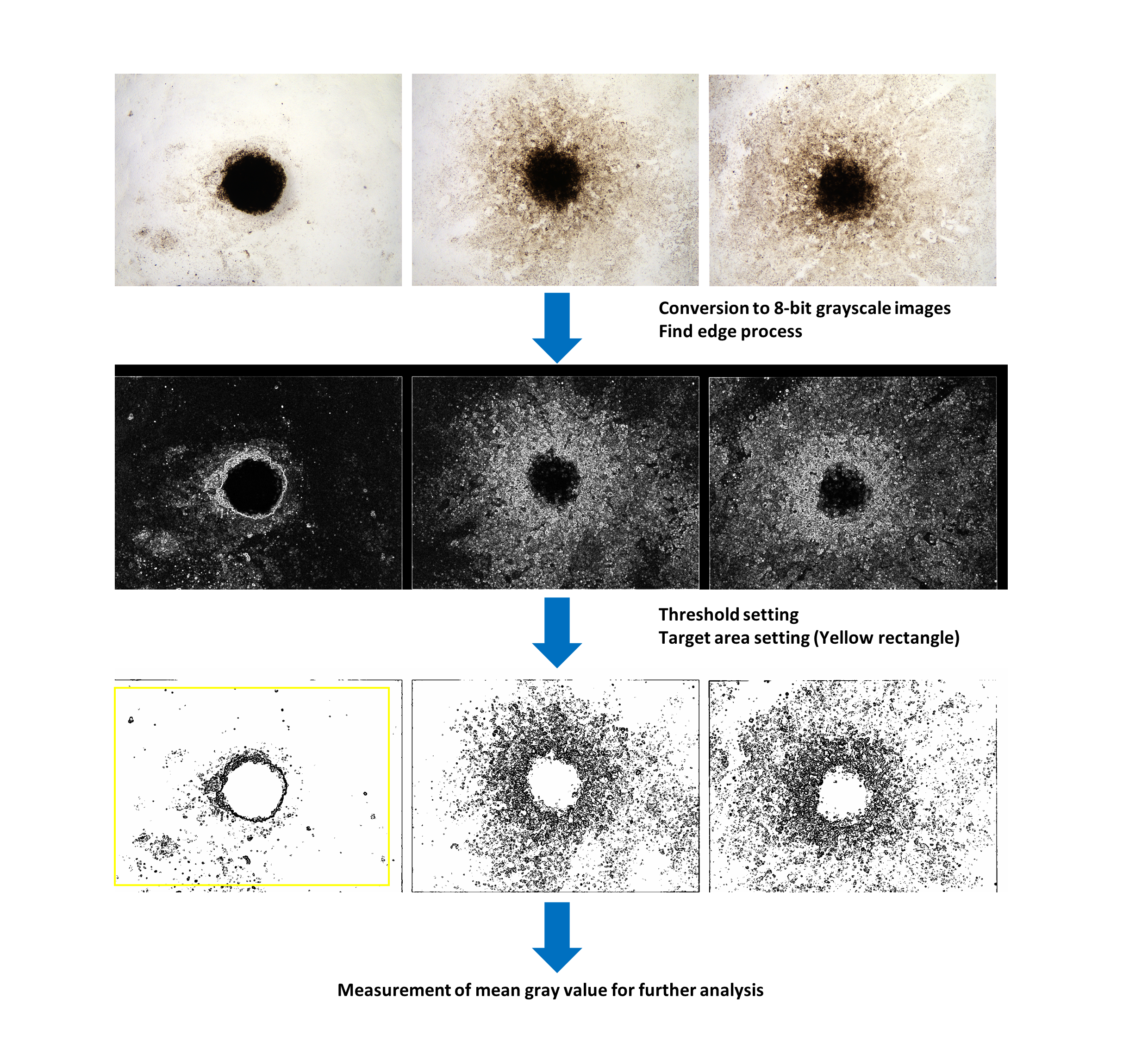
.
