# Supplementary material for: Tissue engineering RPE sheet derived from hiPSC-RPE cell spheroids supplemented with Y-27632 and RepSox
Source: J Biol Eng. 2024 Jan 16;18:7. doi: 10.1186/s13036-024-00405-8 (PMC10790375; doi:10.1186/s13036-024-00405-8)
Supplement: Supplementary file 3 — Additional file 3: Table S1. List of primary antibodies. Table S2. The top 15 KEGG signaling pathways. Table S3. The top 15 GO biological process terms. [file 13036_2024_405_MOESM3_ESM.doc]

| **Antibodies** | **Species** | **Supplier** | **Catalog Number** |
| --- | --- | --- | --- |
| ZO1 | rabbit | Proteintech | 21773-1-Ap |
| MITF | rabbit | Abcam | Ab122982 |
| BEST | mouse | Abcam | Ab259836 |
| Melanoma | rabbit | Abcam | Ab137078 |
| NCAD | mouse | Proteintech | 66219-1-Ig |
| Vimentin | mouse | Affinity | BF8006 |
| Ki67 | rabbit | Invitrogen | MA5-14520 |
| ECAD | mouse | Affinity | BF0219 |
| CRALBP | rabbit | Bioss | Bs-14044R |

**Table A1.** List of primary antibodies.

| ID | KEGG Pathway | Gene Num | Rich Ratio | Q value |
| --- | --- | --- | --- | --- |
| 4514 | Cell adhesion molecules | 34 | 0.228187919 | 3.17E-05 |
| 4512 | ECM-receptor interaction | 23 | 0.261363636 | 1.57E-04 |
| 4974 | Protein digestion and absorption | 24 | 0.233009709 | 5.56E-04 |
| 4610 | Complement and coagulation cascades | 19 | 0.223529412 | 0.005673788 |
| 5033 | Nicotine addiction | 12 | 0.3 | 0.005673788 |
| 5323 | Rheumatoid arthritis | 20 | 0.215053763 | 0.005673788 |
| 4640 | Hematopoietic cell lineage | 19 | 0.191919192 | 0.033137003 |
| 4360 | Axon guidance | 29 | 0.159340659 | 0.035693096 |
| 4080 | Neuroactive ligand-receptor interaction | 48 | 0.135977337 | 0.035693096 |
| 4725 | Cholinergic synapse | 20 | 0.17699115 | 0.048838627 |
| 260 | Glycine, serine and threonine metabolism | 10 | 0.25 | 0.050497689 |
| 5412 | Arrhythmogenic right ventricular cardiomyopathy | 15 | 0.194805195 | 0.058568589 |
| 4020 | Calcium signaling pathway | 34 | 0.141666667 | 0.067195306 |
| 4670 | Leukocyte transendothelial migration | 19 | 0.166666667 | 0.089154697 |
| 4510 | Focal adhesion | 29 | 0.144278607 | 0.089154697 |

**Table A2.** The top 15 KEGG signaling pathways.

| ID | KEGG Pathway | Gene Num | Rich Ratio | Q value |
| --- | --- | --- | --- | --- |
| GO:0007155 | cell adhesion | 133 | 0.194729136 | 5.19E-17 |
| GO:0030198 | extracellular matrix organization | 61 | 0.23828125 | 1.14E-10 |
| GO:0007399 | nervous system development | 103 | 0.179442509 | 1.37E-10 |
| GO:0007411 | axon guidance | 56 | 0.231404959 | 2.27E-09 |
| GO:0071805 | potassium ion transmembrane transport | 35 | 0.239726027 | 9.86E-06 |
| GO:0007268 | chemical synaptic transmission | 54 | 0.188811189 | 1.03E-05 |
| GO:0002687 | positive regulation of leukocyte migration | 10 | 0.666666667 | 2.23E-05 |
| GO:0007156 | homophilic cell adhesion via plasma membrane adhesion molecules | 37 | 0.221556886 | 2.23E-05 |
| GO:0006811 | ion transport | 97 | 0.146304676 | 2.28E-05 |
| GO:0006813 | potassium ion transport | 33 | 0.232394366 | 2.90E-05 |
| GO:0007157 | heterophilic cell-cell adhesion via plasma membrane cell adhesion molecules | 17 | 0.34 | 1.47E-04 |
| GO:0007613 | memory | 24 | 0.260869565 | 1.61E-04 |
| GO:0006836 | neurotransmitter transport | 21 | 0.28 | 2.19E-04 |
| GO:0007420 | brain development | 51 | 0.17114094 | 2.73E-04 |
| GO:0034220 | ion transmembrane transport | 52 | 0.165605096 | 5.55E-04 |

**Table A3.** The top 15 GO biological process terms.
